# Porphyrin Aggregation under Homogeneous Conditions Inhibits Electrocatalysis: A Case Study on CO_2_ Reduction

**DOI:** 10.1021/acscentsci.4c00121

**Published:** 2024-06-03

**Authors:** Kaitlin L. Branch, Erin R. Johnson, Eva M. Nichols

**Affiliations:** † Department of Chemistry, The University of British Columbia, 2036 Main Mall, Vancouver, British Columbia V6T 1Z1, Canada; ‡ Department of Chemistry, Dalhousie University, 6274 Coburg Road, Halifax, Nova Scotia B3H 4R2, Canada

## Abstract

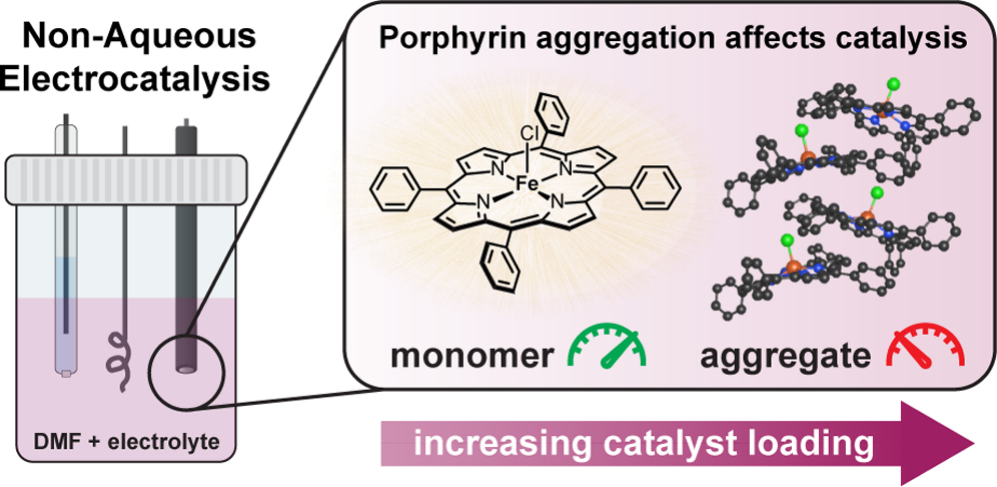

Metalloporphyrins are widely used as homogeneous electrocatalysts for transformations relevant to clean energy and sustainable organic synthesis. Metalloporphyrins are well-known to aggregate due to π–π stacking, but surprisingly, the influence of aggregation on homogeneous electrocatalytic performance has not been investigated previously. Herein, we present three structurally related iron *meso*-phenylporphyrins whose aggregation properties are different in commonly used *N*,*N*-dimethylformamide (DMF) electrolyte. Both spectroscopy and light scattering provide evidence of extensive porphyrin aggregation under conventional electrocatalytic conditions. Using the electrocatalytic reduction of CO_2_ to CO as a test reaction, cyclic voltammetry reveals an inverse dependence of the kinetics on the catalyst concentration. The inhibition extends to bulk performance, where up to 75% of the catalyst at 1 mM is inactive compared to at 0.25 mM. We additionally report how aggregation is perturbed by organic additives, axial ligands, and redox state. Periodic boundary calculations provide additional insights into aggregate stability as a function of metalloporphyrin structure. Finally, we generalize the aggregation phenomenon by surveying metalloporphyrins with different metals and substituents. This study demonstrates that homogeneous metalloporphyrins can aggregate severely in well-solubilizing organic electrolytes, that aggregation can be easily modulated through experimental conditions, and that the extent of aggregation must be considered for accurate catalytic benchmarking.

## Abstract 2

Solution aggregation of metalloporphyrins inhibits electrocatalysis. This complicates intrinsic activity comparisons but offers a new way to modulate activity by controlling aggregate speciation.

## 1 Introduction

The conversion of low-cost and abundant small molecules to value-added products is a growing area of interest for sustainable chemical and fuel production in the context of current environmental challenges.^1^ Electrocatalytic approaches have gained interest due to their ability to operate under mild and tunable conditions by using electricity. Soluble molecular catalysts are often used, because their well-defined and synthetically tunable active sites are amenable to mechanistic inquiry and structure–activity relationships. Specifically, metalloporphyrins have been shown to catalyze an astonishing variety of electrochemical transformations, including the hydrogen evolution reaction (HER),^2−6^ carbon dioxide reduction reaction (CO_2_RR),^7−12^ oxygen reduction reaction (ORR),^13−16^ reduction of nitrogen oxides,^17,18^ and epoxidation^19,20^ and diazidation^21^ of alkenes.

**Figure 1:**
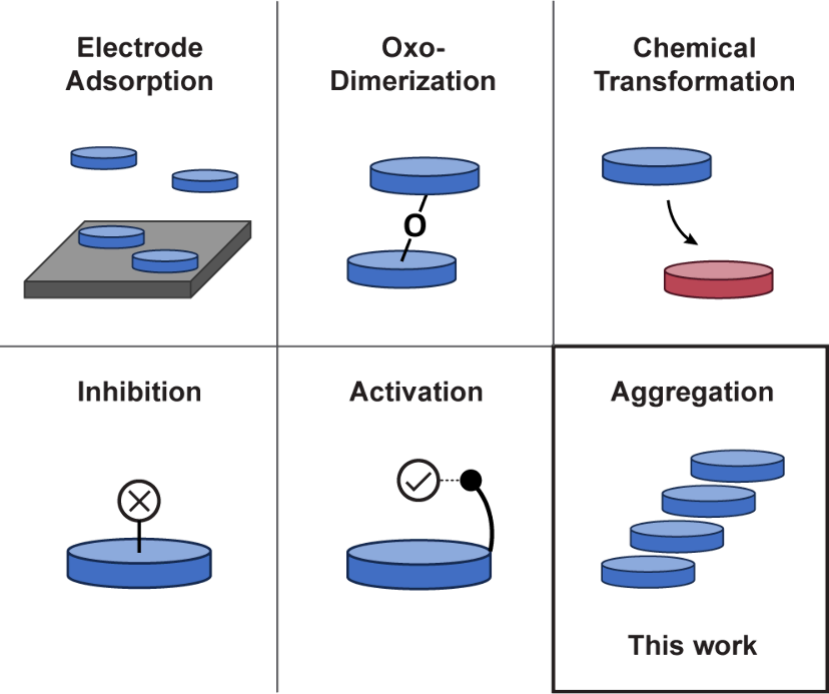
Schematic representation of variable metalloporphyrin speciation in homogeneous electrocatalysis. Colored discs represent generic metalloporphyrin catalysts.

A variety of mechanisms have been identified that alter the speciation—and thus the catalytic performance—of metalloporphyrin electrocatalysts dissolved in a liquid electrolyte. These include adsorption to the electrode surface,^22^ formation of μ-oxo dimers,^23,24^ chemical changes associated with reduction or oxidation,^25−27^ and inhibition^28,29^ or activation^30^ by species present in solution (Figure 1). However, it is nearly always assumed that catalyst concentration is not a critical parameter for electrocatalytic performance (i.e., catalyst concentration dependence is assumed to be first-order), and therefore metrics are usually reported at only one catalyst concentration (typically 0.2–2.0 mM). Herein, we challenge this assumption by showing that metalloporphyrins without extensive steric substitutions are prone to aggregation in solution under the common conditions employed for homogeneous electrocatalysis and demonstrate that solution aggregation has a significant effect on catalytic activity.

It is commonly appreciated that porphyrins are prone to aggregation due to their planar and highly conjugated backbone, which results in favorable π–π stacking that is primarily driven by London dispersion interactions.^31^ The aggregates can have either a J-type (“staircase”) or H-type (“pancake stack”) conformation.^32^ This aggregation phenomenon has been applied in the development of porphyrin-based materials for light harvesting,^33−38^ sensing,^39−41^ nonlinear optics,^42,43^ and photocatalysis.^44−46^ Most solution-phase porphyrin aggregation studies involve biologically relevant aqueous systems, where hydrogen-bonding or ion-pairing effects feature prominently.^47−52^ Porphyrin aggregation in organic solvent has also been reported;^41,53,54^ notably, La Mar and co-workers examined iron tetraphenylporphyrin aggregation by ^1^H NMR spectroscopy in several organic solvents and established a correlation between increasing solvent dielectric constant and more severe aggregation.^55^ Based on this observation, significant aggregation may be expected under conventional homogeneous electrocatalytic conditions due to the large dielectric constants of commonly employed solvents (ε = 36.7 for *N*,*N*-dimethylformamide (DMF) and ε = 37.5 for acetonitrile (MeCN)), which are further increased by the high concentration of supporting electrolyte (typically tetrabutylammonium hexafluorophosphate, TBAPF_6_). Although controlling the extent of aggregation with heterogenized metalloporphyrins^56−59^ and related macrocycles^60−62^ has previously been considered to improve catalytic performance, the extent of solution aggregation and its implications on catalytic performance with homogeneous metalloporphyrins has surprisingly remained mostly unexplored prior to this report (see Supporting Information for a discussion of this limited literature). Here, we address these questions with a focus on iron porphyrins—efficient and selective catalysts for the reduction of CO_2_ to CO^7,29,63−65^—although we contend that many conclusions may be extended to other electrocatalytic reactions.

In the current work, we compare a family of electronically equivalent iron porphyrins that have variable dispersion interaction strengths due to the presence of two, three, or four *meso* phenyl rings. Using UV–visible spectroscopy and dynamic light scattering, we demonstrate that these porphyrins aggregate significantly under electrocatalytically relevant conditions and that the extent of aggregation is correlated to the number of *meso* phenyl rings. Cyclic voltammetry is used to determine the kinetics of electrocatalytic CO_2_ reduction as a function of the catalyst concentration. An *inverse* order in catalyst concentration is seen in the rate laws for all three porphyrin catalysts, and bulk electrolysis experiments confirm that the extent of aggregation directly influences the current density and amount of CO generated. To further clarify the relationship between catalyst aggregation and activity, we induce metalloporphyrin disaggregation upon the addition of pyrene as a competitive aggregator or by abstraction of the axial chloride with AgPF_6_; in each case, the CO_2_ reduction activity is increased. These findings are generalized by surveying other simple metalloporphyrin complexes with varying metal identities and ligand substitutions. Evidence of aggregation is broadly observed, thereby highlighting the generality of this effect; as such, the findings presented here likely extend to other homogeneous metalloporphyrin-catalyzed transformations. This work underscores the importance of checking for aggregation with homogeneous porphyrin electrocatalysts to properly benchmark activity and offers new directions to improve performance by tailoring catalyst structure and operating conditions to minimize aggregation.

## 2 Results and Discussion

### 2.1 Selection of Iron Porphyrins

**Figure 2:**
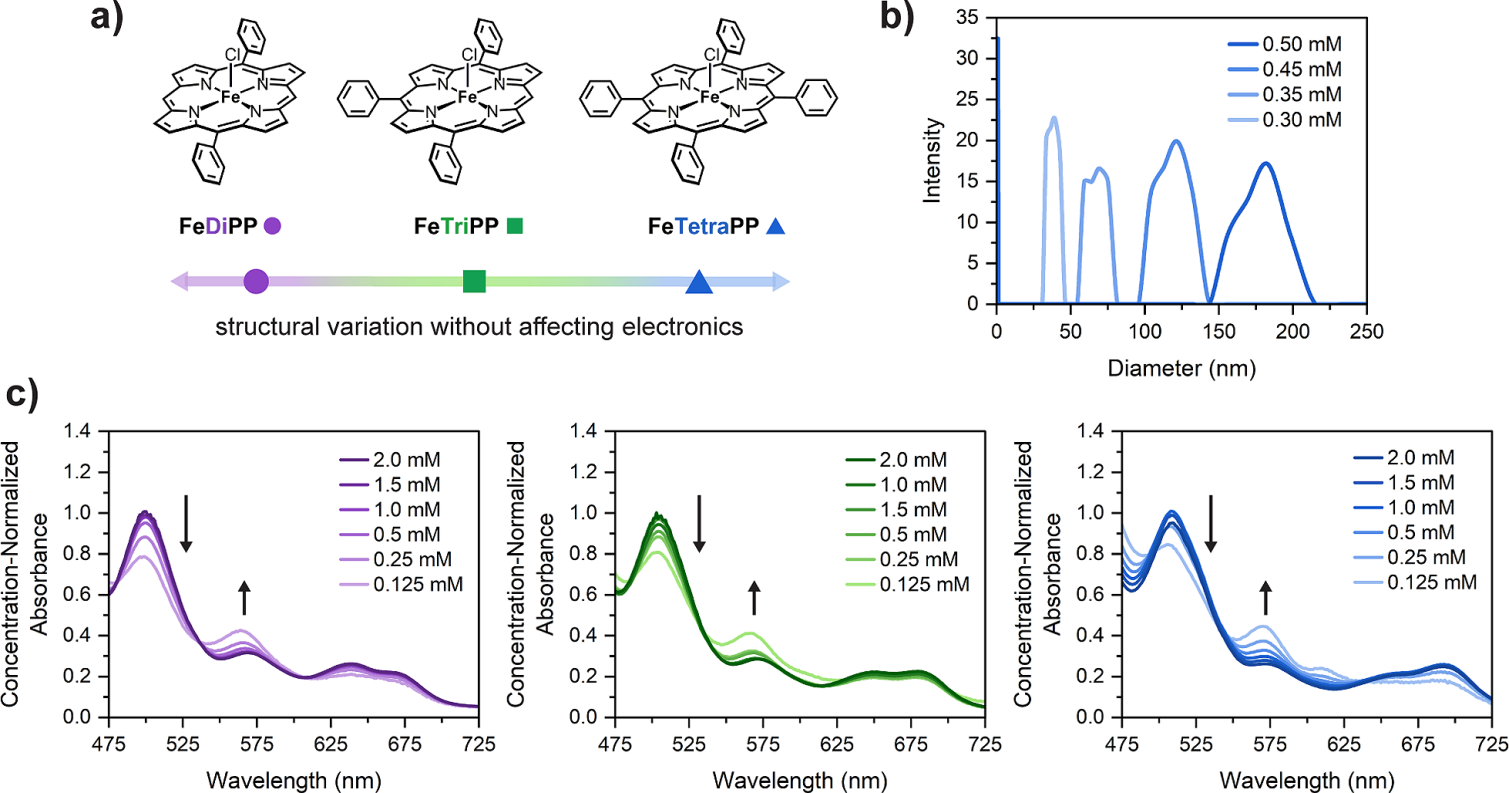
(a) Structures of iron(III) *meso*-phenylporphyrin catalysts studied in this work. (b) Dynamic light scattering (DLS) analysis of **FeTetraPP** aggregates at variable concentrations. Conditions: 0.5–0.3 mM porphyrin as indicated, 0.1 M TBAPF_6_ in DMF. (c) UV–vis absorption spectra as a function of the catalyst concentration. Concentration-normalized spectra (absorbance divided by catalyst concentration) of the Q-band region at 2.0–0.125 mM of **FeDiPP** (left), **FeTriPP** (middle), and **FeTetraPP** (right). Arrows depict the spectral changes upon dilution. Conditions: indicated catalyst concentration, 0.1 M TBAPF_6_ in DMF, 1 mm path length.

In order to correlate electrocatalytic activity with aggregation severity, we sought a series of iron porphyrin complexes with similar electronics (to prevent ambiguity arising from electronic scaling effects^66^) but variable tendency to form solution aggregates. Given the known importance of dispersive interactions in porphyrin π–π stacking,^31^ we hypothesized that variation of the number of *meso* phenyl substituents around the porphyrin ligand would result in different aggregation behavior. Conveniently, the Hammett parameters (σ_p_) for phenyl and proton substituents are nearly identical,^67^ suggesting that all porphyrins in this series should have similar reduction potentials regardless of the number of *meso* phenyl rings. We therefore targeted the iron(III) chloride complexes of 5,15-diphenylporphyrin (**FeDiPP**), 5,10,15-triphenylporphyrin (**FeTriPP**), and 5,10,15,20-tetraphenylporphyrin (**FeTetraPP**), as illustrated in Figure 2a. The fully substituted porphyrin complex **FeTetraPP** is well-known as an electrochemical CO_2_ reduction catalyst, while complexes with *meso*-H substituents like **FeDiPP** and **FeTriPP** have not been previously investigated in homogeneous CO_2_ electrocatalysis to our knowledge.

The free-base porphyrin ligands DiPP and TetraPP were obtained commercially, while TriPP was prepared by addition of phenyllithum to DiPP, followed by reoxidation. Metalation with FeCl_3_ afforded the desired iron(III) *meso*-phenylporphyrin chloride complexes. Additional details regarding the synthesis and characterization are provided in the Supporting Information.

### 2.2 Characterization of Iron Porphyrin Aggregates under Conditions Used in Homogeneous Electrocatalysis

UV–visible (UV–vis) spectroscopy is a simple yet powerful tool for investigating aggregation of metalloporphyrins whereby peak shifts and deviations from Beer’s law correlate with the extent of aggregation in solution.^47,48,68,69^ To investigate aggregation under catalytically relevant conditions, UV–vis spectra at variable iron porphyrin concentrations (2.0–0.125 mM) were collected in DMF containing a supporting electrolyte (0.1 M TBAPF_6_). These high porphyrin concentrations necessitated the use of a short-path cuvette (1 mm) and were limited to the low-intensity Q bands of the iron porphyrins. Nevertheless, after normalizing the spectra for porphyrin concentration, a series of alternating-intensity changes of the Q bands were observed for each catalyst (Figure 2c), suggesting a change in solution speciation as a function of catalyst concentration within a range relevant to typical catalyst loadings in homogeneous electrocatalysis. It is possible for Q-band changes to originate from speciation changes other than aggregation (see SI and Figure S3), so further investigations were conducted to verify this interpretation (*vide infra*).

UV–vis spectroscopy has been extensively used to characterize the structure of porphyrin aggregates as either J-type (staircase) or H-type (face-to-face) based on a respective red- or blue-shift of the Soret band with increasing concentration.^32,68,69^ The Soret absorbance was saturated at catalytically relevant mM concentrations due to its large extinction coefficient. However, aggregation was still observed at concentrations over an order of magnitude more dilute than those typically used in electrocatalysis (<0.1 mM), as all three catalysts display a red-shift in the Soret band with increasing catalyst concentration (Figure S4), supporting assignment as a J-aggregate structure. **FeDiPP** displays the most pronounced red-shift followed by **FeTriPP** and finally **FeTetraPP**, which required additional dilutions to observe a shift. This suggests that the aggregation state of **FeDiPP** changes the most significantly over this concentration range. Overall, these results indicate a difference in aggregation severity across the series as opposed to a difference in aggregate structure type (H- vs J-type).

The formation of solution aggregates under catalytically relevant conditions was further confirmed by dynamic light scattering (DLS). Solutions of **FeTetraPP** (0.50–0.30 mM) in DMF containing supporting electrolyte (0.1 M TBAPF_6_) were examined; higher porphyrin concentrations were not used due to significant absorption of the incident light. Aggregates with diameters between 25 and 225 nm were detected across this concentration range (Figure 2b); however, these sizes are not directly representative of the true size of J-aggregates since DLS assumes spherical particles. Nevertheless, these results show that as porphyrin concentration is increased, both the average size and the size distribution of the aggregates increase (Figure 2b). These results clearly demonstrate the formation of solution aggregates under conditions commonly employed in electrocatalysis and show that the extent of aggregation is highly dependent on porphyrin concentration.

### 2.3 Electrochemistry of Variably Substituted Iron *meso*-Phenylporphyrin Catalysts under Argon

**Figure 3:**
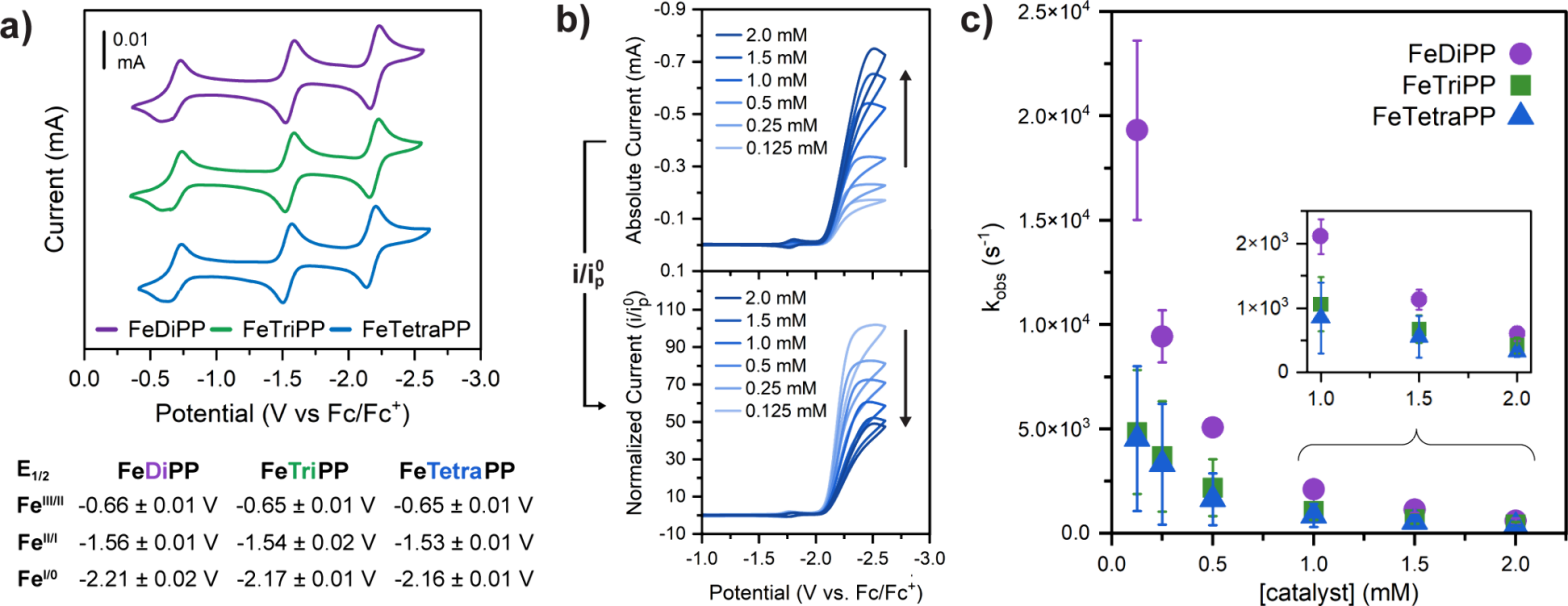
Electrochemical and catalytic studies. (a) Cyclic voltammograms of **FeDiPP**, **FeTriPP**, and **FeTetraPP**. Conditions: 1.0 mM catalyst, 0.1 M TBAPF_6_ in Ar-saturated DMF using a glassy carbon electrode, and 100 mV/s scan rate. Below: mean reduction potentials (vs Fc/Fc^+^) from three independent CV measurements; errors are 1 standard deviation. (b) Catalyst concentration-dependent CVs (top plot) and CVs normalized by *i*_p_^0^, the peak current of the Fe^II/I^ peak (bottom plot) of **FeTetraPP**. (c) Mean observed rate constants (*k*_obs_) as a function of catalyst concentration for each iron porphyrin based on three independent CV measurements; error bars represent 1 standard deviation. Conditions: indicated catalyst concentration, 250 mM PhOH, 0.1 M TBAPF_6_ in CO_2_-saturated DMF, and 100 mV/s scan rate.

We first evaluated the electrochemical behavior of each iron porphyrin under argon in DMF at a standard concentration of 1.0 mM. It is worth noting that all iron porphyrin complexes are fully soluble at this concentration. The cyclic voltammograms (CVs) show three consecutive chemically and electrochemically reversible single-electron reduction events corresponding to the formal Fe^III/II^, Fe^II/I^, and Fe^I/0^ redox couples (Figure 3a). The reduction potentials across the series are largely invariant when considering experimental error (Figure 3a), as predicted based on the Hammett parameters for phenyl vs H (see Section 2.1). This electronic similarity enables direct correlations between activity, structure, and aggregation state without convolution from well-established electronic scaling relationships.^66^ The possibility of catalyst adsorption on the working electrode was ruled out by examining the peak current as a function of the square root of the scan rate: a linear relationship was observed for all concentrations of each catalyst at all iron redox states (Figure S5), indicative of a diffusional process without substantial electrode adsorption. Additionally, no pre-waves are observed in the argon CVs at any concentration (Figure S6), again suggesting that the catalysts are indeed homogeneous.^22^ To further exclude the likelihood of catalyst adsorption, the working electrode was rigorously polished after every CV. Finally, we calculated diffusion coefficients at a range of catalyst concentrations from the variable scan rate CVs according to the Randles–Ševčík equation and attempted to correlate this to the extent of aggregation; however, no clear trend was observed (Figure S7).

### 2.4 Electrocatalytic CO_2_ Reduction Is Inhibited Due to Porphyrin Aggregation

Having established that all three iron porphyrins exhibit solution aggregation under electrochemically relevant conditions, we used CO_2_ reduction as a case study to understand the implications of this aggregation on the electrocatalytic performance. Under a CO_2_ atmosphere with phenol (PhOH) as the exogenous acid source, CV traces show large current responses at the formal Fe^I/0^ couple, consistent with the qualitative expectation that these complexes are active CO_2_ reduction catalysts (Figures 3b and S14). For a more quantitative approach, foot-of-the-wave analysis (FOWA)^29^ was used to extract observed rate constants, *k*_obs_, from the CV responses (see Supporting Information for details). First, *k*_obs_ was measured at variable PhOH concentrations (50–1000 mM): all catalysts show that the PhOH dependence is initially first-order followed by saturation at higher PhOH concentrations (Figure S8). Based on these results, we selected 250 mM PhOH as a suitable concentration to compare the activity of all catalysts within the linear acid-dependence regime.

CVs under a saturated CO_2_ atmosphere in the presence of 250 mM PhOH were collected at six different catalyst concentrations, ranging from 0.125 to 2.0 mM. A comparison of current responses on an absolute scale (i.e., non-normalized CVs) shows increasing peak currents with increasing catalyst concentrations (Figure 3b, top) and may preliminarily suggest a positive order dependence on catalyst concentration. However, when the current responses are appropriately normalized by the amount of iron porphyrin in solution (determined based on *i*_p_^0^, the porphyrin peak current in absence of substrate; the Fe^II/I^ couple is used because this redox feature is the least sensitive to small changes in solution composition and thus the most accurate), the current responses *decrease* as the catalyst concentration is increased (Figure 3b, bottom). FOWA was used to determine *k*_obs_ at each catalyst concentration, and an *inverse* dependence is seen for all three porphyrins, including the well-studied **FeTetraPP** (Figures 3c, S9, Table S1). This relationship clearly indicates that the catalysts become inhibited at higher porphyrin concentrations, where aggregation predominates. The severity of inhibitive aggregation correlates with the number of *meso* phenyl groups: the kinetics of **FeTriPP** and **FeTetraPP** are indistinguishable within error, but the kinetics of **FeDiPP** increase significantly at lower catalyst concentrations, plausibly as a result of **FeDiPP**’s smaller propensity to form aggregates.

Interestingly, while *k*_obs_ for electrocatalytic CO_2_ reduction inversely correlates with catalyst concentration, the peak current (*i*_p_^0^) for single-electron transfer to the catalyst does not show the same effect. That is, *i*_p_^0^ for all three catalysts increases linearly with catalyst concentration as expected based on the Randles–Ševčík equation (Figure S10). This is good evidence that the number of *redox-active* porphyrins in the diffusion layer scales linearly with solution concentration, whereas the number of *catalytically active* porphyrins decreases exponentially. These findings are consistent with the formation of solution aggregates that maintain electronic conductivity through the assembly but where only a fraction of porphyrins—likely those on the ends of the aggregates—are catalytically active (Figure S11).

Finally, we sought to explore the implications of catalyst aggregation on the CO_2_ concentration dependence in the rate law. CVs were recorded with variable concentrations of CO_2_ by sparging with argon/CO_2_ mixtures prepared with precision mass flow controllers. The observed trend in the CO_2_ dependence is also influenced by the chosen catalyst concentration. When the catalyst concentration is low (0.25 mM), the expected first-order dependence on CO_2_ is observed (Figure S12a). At 1.0 mM catalyst—where aggregation is more severe—the CO_2_ dependence displays linear behavior only until ca. 60% CO_2_ and is then followed by a region where the kinetics plateau or decrease (Figure S12b). We note that this deviation from linearity at 1.0 mM **FeTetraPP** was not observed in analogous experiments performed at a higher phenol concentration (500 mM vs 250 mM), both in a previous report^70^ and in our hands (Figure S13). It is evident that the rate laws with this family of catalysts exhibit previously undiscussed complexities, wherein the concentrations of all reaction participants are not always independent. The role of phenol on porphyrin aggregation state will be discussed further in Section 2.6.

### 2.5 Product Selectivity and Concentration-Dependent Bulk Performance

The results presented thus far show a clear correlation between inhibited catalyst kinetics (by CV) and increased porphyrin aggregation (by UV–vis and DLS). In order to provide insight into how aggregation influences catalyst stability, selectivity, and bulk performance, we performed preparative-scale electrolysis with the series of iron porphyrin catalysts at variable catalyst concentrations.

**Figure 4:**
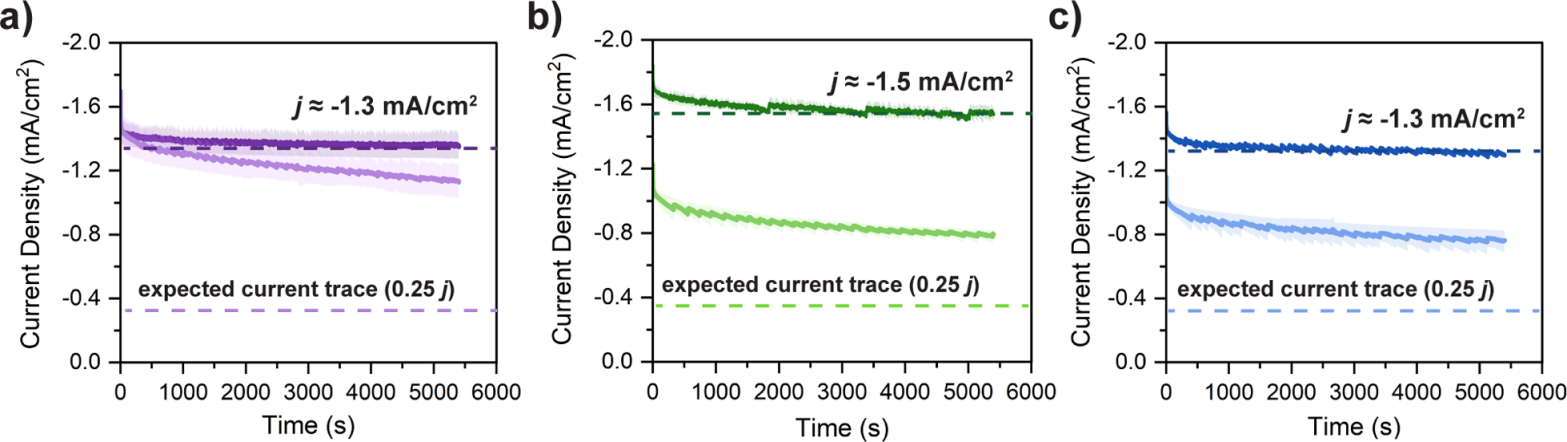
Controlled potential electrolysis experiments of (a) **FeDiPP**, (b) **FeTriPP**, and (c) **FeTetraPP**. Conditions: 1.0 mM (dark traces) or 0.25 mM (light traces) catalyst concentration, 100 mM PhOH, 0.1 M TBAPF_6_ in CO_2_-saturated DMF, 90 min at ∼−2.2 V vs Fc/Fc^+^. Performed in duplicate; average currents are reported, and shaded regions represent the standard error when larger than the data trace itself. Dark dashed lines represent average current density at 1.0 mM porphyrin and light dashed lines show one fourth of this current density.

Controlled potential electrolysis (CPE) experiments were performed in a custom-built, gastight electrolysis cell, and products were detected by headspace analysis on a gas chromatograph (see SI for details). First, experiments performed at 1.0 mM catalyst concentration showed that all three catalysts achieve comparable current densities, stabilities, and total charge passed over a 90 min electrolysis (Figure 4, dark traces). CPE experiments were then repeated at 0.25 mM catalyst to investigate concentration-dependent effects; here, all catalysts exhibit significantly higher current density profiles (Figure 4, light traces) than would be expected based on dilution alone (shown by the lighter dashed lines depicting one fourth of the current density observed at 1.0 mM catalyst). These findings are consistent with the previously presented studies of *k*_obs_ determined by CV, both in the inverse dependence on catalyst concentration and in the trend between the catalyst structure and activity. That is, the activity achieved at the dilute catalyst concentration is largest for **FeDiPP**, whereas **FeTriPP** and **FeTetraPP** achieve similar current density profiles. Product selectivity was not found to depend on aggregation state or the number of phenyl substituents, with a Faradaic efficiency of >77% for carbon monoxide (CO) in all cases and no hydrogen detected (Table S2). No evidence of catalyst decomposition was found through CV or UV–vis on post-electrolysis solutions (Figures S17 and S18), demonstrating—for the first time to our knowledge—that molecular porphyrin complexes bearing unsubstituted *meso* positions are reductively stable under CO_2_ reduction conditions.

Overall, these CPE results demonstrate that aggregation significantly influences the bulk CO_2_ reduction performance of the porphyrin catalysts. For instance, in the case of **FeDiPP**, both catalyst loadings yield almost identical current profiles, meaning that nearly 75% of the catalyst at 1 mM is inactive. Additionally, it is evident that the catalytic activity is strongly influenced by the chosen operating conditions, including catalyst concentration. We therefore stress that differences in catalytic performance may be mistakenly attributed to inherent activity when they are actually driven by differences in the aggregation state.

### 2.6 Perturbing Porphyrin Aggregation State Influences Catalytic Activity

The data reported thus far show that the porphyrin concentration influences the extent of aggregation, which in turn affects the CO_2_ reduction activity. This begs the question of whether aggregation status can be perturbed (e.g., via chemical additives or an external stimulus) and, if so, how operating conditions can be tailored to avoid aggregation or even restore activity of a highly aggregated catalyst.

**Figure 5:**
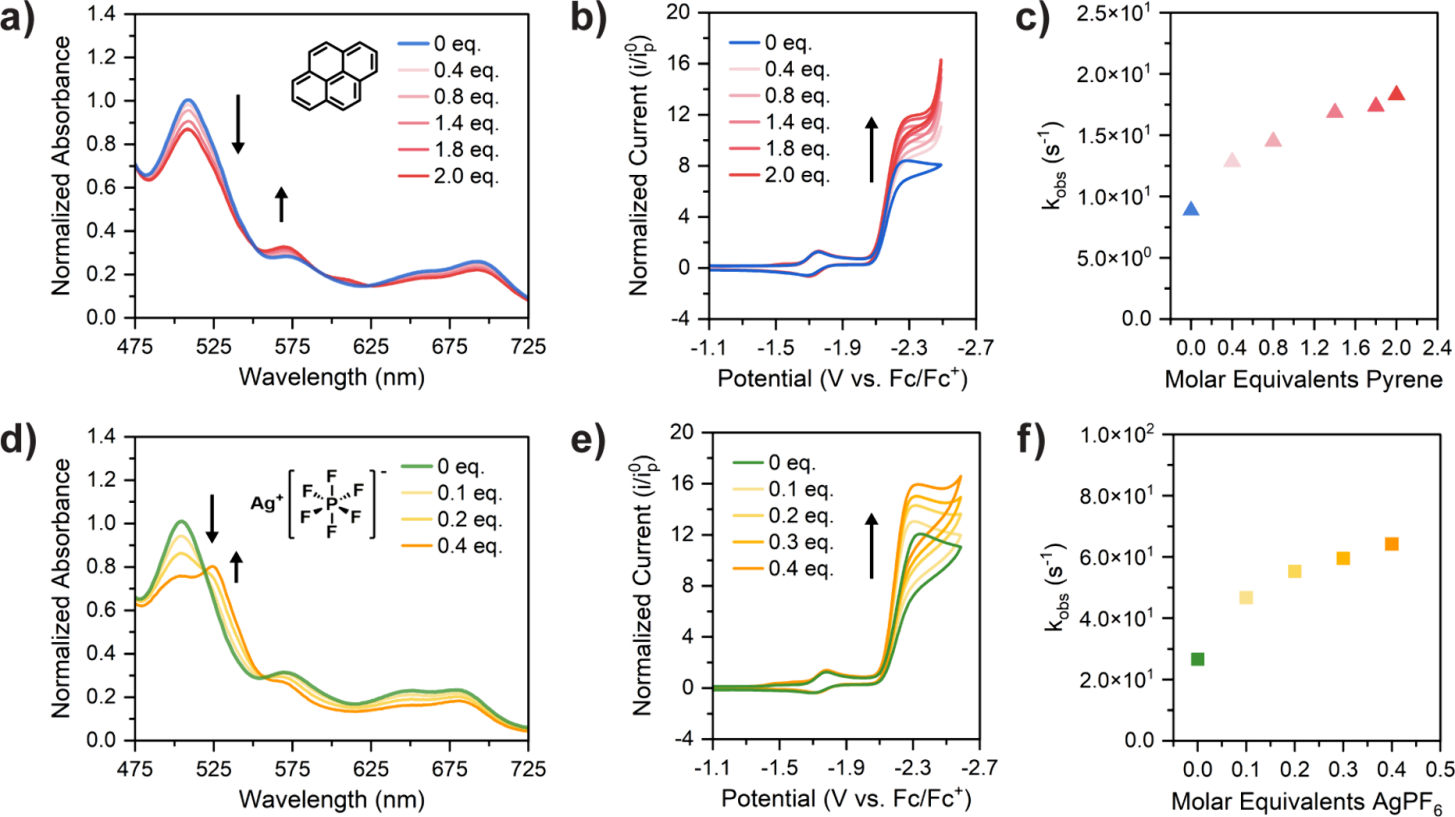
(a–c) Titration of 0–2 molar equiv of pyrene to 1.0 mM **FeTetraPP**: (a) UV–vis spectra of the Q-band region upon titration. Conditions: 0.1 M TBAPF_6_ in DMF, 1 mm path length. (b) CVs upon titration. (c) Observed rate constants (*k*_obs_) at each pyrene concentration. Conditions: 10 mM PhOH, 0.1 M TBAPF_6_ in CO_2_-saturated DMF, and 100 mV/s scan rate. (d–f) Titration of 0–0.4 molar equiv of AgPF_6_ to 0.5 mM **FeTriPP**: (a) UV–vis spectra of the Q-band region upon titration. Conditions: 0.1 M TBAPF_6_ in Ar-saturated DMF, 1 mm path length. (b) CVs upon titration. (c) Observed rate constants (*k*_obs_) for each titration of AgPF_6_. Conditions: 10 mM PhOH, 0.1 M TBAPF_6_ in CO_2_-saturated DMF, 100 mV/s scan rate.

We first set out to alter the aggregation state by simply disrupting the interactions within the porphyrin assemblies using a chemical additive as a proof of concept. Pyrene was identified as a planar, conjugated molecular additive, which we hypothesized could competitively engage in π–π stacking and induce disaggregation of the metalloporphyrin assemblies. UV–vis spectra of 1.0 mM **FeTetraPP** with added pyrene show Q-band intensity changes (Figure 5a) that are analogous to those observed in the concentration-normalized spectra (Figure 2c), showing that pyrene is indeed acting as a disaggregating agent in our system. This pyrene titration was then performed in CO_2_ reduction electrocatalysis; here, the catalytic currents and resulting *k*_obs_ values increase upon each addition of pyrene (Figure 5b,c), thereby further establishing the correlation between reduced catalyst aggregation and an improvement in the catalytic performance. In bulk electrolysis with 1 molar equivalent of pyrene, an increase in the charge passed was observed (Figure S20), again demonstrating disaggregation. We additionally explored whether phenol (PhOH), commonly chosen as an exogenous acid in electrocatalytic CO_2_RR, could similarly act as a disaggregating additive. No significant UV–vis spectral changes were observed upon PhOH additions (0–500 mM) to a concentrated porphyrin solution, suggesting a negligible effect on the porphyrin aggregation state (Figure S21). These results suggest that the interdependencies between terms in the rate law—notably observed in the different CO_2_ dependence behavior at 250 vs 500 mM PhOH (Figures S12 and S13)—do not likely arise from PhOH-induced changes in aggregation state, although the possible role of PhOH-derived species formed *in situ* was not explored.

We next considered the role of axial ligation on aggregate structure, specifically whether the identity of this ligand—chlorido vs solvato—in the isolated iron(III) porphyrin influences aggregation state. For iron(III) porphyrins, dimer structures have been proposed and the identity of the axial halide was found to alter the propensity to associate.^55^ Additionally, chloro-manganese(III) porphyrins have been shown to act as a “chain-capping” group to control the size of divalent porphyrin assemblies.^71^ This suggests that porphyrin complexes bearing axial ligands may be less prone to aggregation, although this study was performed in a nonpolar organic solvent with a limited relevance to electrocatalysis. We explored abstraction of the axial chloride through titrations with AgPF_6_ and observed the evolution of UV–vis features attributable to the iron(III) DMF-bound porphyrin^72^ (Figure 5d). These UV–vis changes are also similar to those observed upon porphyrin dilution and pyrene addition, suggesting that disaggregation accompanies the change in axial ligand identity from chloride to DMF. We propose that the change in electrostatics and structure upon going from a neutrally charged Fe(III) porphyrin with an inner-sphere chloride to a cationic Fe(III) porphyrin with an axial DMF underlies the disaggregation. Repeating this chloride abstraction titration under CO_2_ reduction conditions shows an increase in electrocatalytic activity (Figure 5e,f), providing additional support for the proposal that chloride abstraction results in porphyrin disaggregation. Interestingly, a previous report observed the same increase in CO_2_ reduction activity with **FeTetraPP** in acetonitrile electrolyte following abstraction of the axial chloride, but attributed this change to increased porphyrin solubility and an altered reaction mechanism without discussion of changes in speciation/aggregation state.^73^ Finally, to elaborate on the role of axial ligation in self-assembly, we repeated the concentration-normalized UV–vis dilution experiment in the presence of excess chloride (TBACl) to shift the equilibrium in favor of the chloride-bound complex; this prevents disaggregation associated with dilution (Figure S23), showing that the presence of the axial chloride strongly favors aggregate formation and suggesting an interdependence between catalyst dilution, equilibrium chloride/DMF binding, and disaggregation. Together, these results imply that the axial ligand has a significant influence on the self-assembly of porphyrin complexes, a finding that may offer interesting ways to tune the catalyst activity via speciation changes.

Having so far only discussed aggregation of the isolated iron(III) precatalysts, we next used UV–vis spectroelectrochemistry to probe whether the propensity to aggregate is dependent on redox state. Several previous reports investigated aggregation of divalent metalloporphyrins in organic solvents from the perspective of supramolecular chemistry and photophysics,^41,54,74^ but little information is available on aggregation behavior of lower-valent species (which would bear overall anionic charges). Concentration-normalized UV–vis spectra of the Q-band region were collected through a Pt mesh working electrode while simultaneously applying potentials corresponding to each redox state of interest. The spectra of the formal Fe(II), Fe(I), and Fe(0) species display concentration-dependent features consistent with aggregation (Figure S24). Overall, these spectroelectrochemical experiments suggest that the aggregation inhibition effect observed in electrocatalysis likely arises from concurrent aggregation of multiple porphyrin species at various redox states within the catalytic cycle (e.g., precatalyst, resting state, and/or active species) (Figure S25).

### 2.7 Modeling the Structures and Energies of Iron Porphyrin Aggregates

Periodic boundary calculations were performed to investigate aggregate structures and energies for each *meso*-phenyl iron(III) porphyrin (see SI for details). Aggregates were constructed from chloride-bound porphyrin monomers, which were established as the predominant aggregating species by chloride-abstraction UV–vis titrations. All spin states were assigned as *S* = 5/2 according to previous reports on chloride-bound **FeTetraPP**,^75^ with the assumption that the number of *meso*-phenyl groups does not strongly influence spin. For each complex, three different aggregate conformations were initially examined: two structures with axial chloride ligands oriented antiparallel and a structure with axial chloride ligands oriented parallel (Figure S26). Of these different conformations, the latter structure resulted in the most stable aggregates with the greatest binding energies (Table S3). The parallel orientation of axial chloride ligands likely minimizes electrostatic repulsion, and the staircase conformation of this structure agrees with the J-type assignment made through observed red-shifts in the Soret band by UV–vis investigations. As such, this staircase structure was chosen as the model for our porphyrin aggregates.

**Figure 6:**
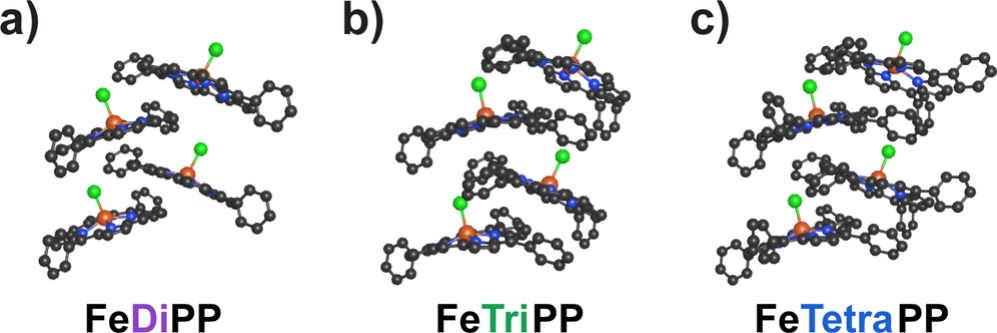
Model of the J-aggregate structure of (a) **FeDiPP**, (b) **FeTriPP**, and (c) **FeTetraPP** with chloride ligands in a parallel orientation. Hydrogen atoms have been removed for clarity.

With the optimized aggregate structures for each catalyst (Figure 6), the binding energies and HOMO–LUMO gap shifts were then calculated and compared with experimental results. The **FeDiPP** aggregate was found to have the weakest binding energy, while aggregates of **FeTriPP** and **FeTetraPP** are more stable with comparable binding energies (Table 1). The phenyl groups in the aggregates appear to rotate slightly in order to interact with the porphyrin ring of neighboring molecules, suggesting that increasing the number of phenyl rings may contribute to favorable stacking interactions and increased aggregate stability. This trend in aggregate stability across the series correlates with the catalyst concentration-dependent CO_2_ reduction electrocatalysis: the observed rate constants for **FeTriPP** and **FeTetraPP** at each concentration are indistinguishable within error, whereas the kinetics of **FeDiPP** are similar in the high-concentration regime but get increasingly large at lower concentrations (Figure 3c). This observation can be rationalized by considering the aggregate stability across the series, where the weaker aggregate binding of **FeDiPP** may result in more appreciable disaggregation upon dilution, whereas the more stable aggregates of **FeTriPP** and **FeTetraPP** persist, even at lower catalyst loadings. The red-shifted Soret bands observed in UV–vis (Figure S4) are reproduced in the aggregate models that show a calculated decrease in the HOMO–LUMO energy gap upon aggregate formation (Table 1). The magnitudes of the calculated gap shifts across the series are also consistent with experimental red-shifts of the Soret band, where **FeDiPP** and **FeTriPP** show similar and more severe shifts compared to **FeTetraPP** (Figure S4). Together, these computations demonstrate the differences in aggregate stability across the catalyst series and offer insight into the observed structure-dependent catalytic performance. We note, however, that these calculations neglect explicit solvent/electrolyte interactions and thus do not account for the role of these species on the structure and stability of the porphyrin aggregates.

**Table 1:** Summary of the Calculated Energies for Modeled Porphyrin Aggregate Structures

catalyst	aggregate binding energy^a^ (eV)	HOMO–LUMO gap shift (eV)
**FeDiPP**	–0.838	–0.090
**FeTriPP**	–1.035	–0.096
**FeTetraPP**	–1.099	–0.054

a Aggregate binding energy per molecule.

### 2.8 Survey of Additional Metalloporphyrins: Structural Factors Affecting Self-Assembly

The above results document the relationship between the iron porphyrin structure and severity of aggregation in the context of CO_2_ reduction. In order to assess whether this documented aggregation effect is generalizable to other electrocatalytic reactions, a survey of various metalloporphyrin complexes in DMF or MeCN electrolyte was undertaken using concentration-normalized UV–vis studies as a metric for aggregation severity.

First, to further explore aggregation properties as a function of catalyst structure, we surveyed additional simple iron porphyrin catalysts with different substitutions. Methyl and methoxy substitutions on the *meso*-phenyl rings both appear to reduce the severity of aggregation to some extent, whereas perfluorination of the phenyl groups does not (Figure S27). We previously reported that iron porphyrin catalysts substituted with a second coordination sphere pendent group oriented above the porphyrin plane demonstrate agreement with a first-order catalyst dependence over a comparable concentration range, suggesting that pendent groups may be sufficiently large to prevent significant catalyst aggregation.^11,76^ The orientation of these groups also appears to be important, as first-order behavior is no longer observed when the pendent group is instead oriented in the plane of the porphyrin.^30^ Taken together, these results suggest that simple functionalization of the porphyrin does not fully prevent aggregation, whereas incorporation of second coordination sphere groups orthogonal to the porphyrin plane may play an additional steric role by reducing the propensity for aggregation.

The increased solubility of substituted iron tetraphenylporphyrins permitted investigations into aggregation in MeCN electrolyte, another commonly used solvent for electrocatalysis. Evidence of aggregation was observed in UV–vis under these conditions (Figure S28), highlighting that the aggregation effect is likely not unique to DMF solvent. As such, the possibility of aggregation for metalloporphyrin catalysts should be investigated, regardless of the chosen operating solvent and conditions.

We additionally surveyed the aggregation of several metalloporphyrins with divalent metal ions to further evaluate the role of coordination geometry and electronic structure on porphyrin aggregation. Concentration-normalized UV–vis spectra in the Q-band region were collected for cobalt-, nickel-, copper-, and zinc-tetraphenylporphyrins. Under conditions relevant to electrocatalysis, these complexes (with the exception of the zinc complex) display concentration-dependent changes in their absorption spectra (Figure S29), suggesting that the aggregation effect extends to most divalent metalloporphyrins. Surprisingly, the severity of aggregation appears to be highly dependent on metal identity, despite the structural similarity across the series; the nickel and copper complexes show the most pronounced spectral changes followed by the cobalt complex, whereas the zinc complex shows no evidence of aggregation under these conditions. Additionally, the free-base **DiPP**, **TriPP**, and **TetraPP** ligands were surveyed and do not show evidence of aggregation in concentration-normalized spectra (Figure S30), further highlighting the involvement of metalation in the aggregation properties of porphyrins.

### 2.9 Discussion of Factors Influencing Aggregation and Implications for Electrocatalysis

In summary, we outline several factors that affect the aggregation state—and correspondingly the activity—of metalloporphyrin electrocatalysts under homogeneous conditions:

1. *Catalyst loading*: Lower porphyrin concentrations (<1 mM) reduce the extent of aggregation.
2. *Catalyst structure*: Decreasing the number of *meso* phenyl rings on the catalyst (**FeTetraPP** vs **FeTriPP** vs **FeDiPP**) decreases the strength of the London dispersion interactions that lead to porphyrin aggregation; **FeDiPP** forms the least stable aggregates and displays the highest catalytic activity at low concentration. Bulky substitutions on the *meso* phenyl rings appear to reduce the severity of aggregation.
3. *Axial ligation*: Abstraction of the axial chloride ligand (for iron(III) porphyrins) induces disaggregation, while excess chloride favors aggregation. The presence and identity of axial ligands will likely be a key design parameter for minimizing aggregation.
4. *Redox state*: Spectroelectrochemical evidence shows that aggregation occurs at a range of porphyrin redox states, regardless of overall complex charge.
5. *Metal identity*: Several divalent metalloporphyrins (Co, Ni, Cu) show UV–vis evidence of aggregation, although the type and severity of aggregation may be variable.

Importantly, differences in catalyst structure and/or chosen operating conditions influence the aggregation state, a finding that has direct implications on meaningful catalyst comparisons. Evaluating the electrocatalytic activity for two porphyrins may lead to different conclusions regarding their intrinsic activity depending on the extent to which each catalyst is aggregated. It is therefore necessary to evaluate performance at a range of catalyst concentrations in order to assess the extent of aggregation. With these conditions identified, catalyst activity can be meaningfully compared under the ideal conditions, where there is no aggregation or where aggregation is minimized.

## 3 Conclusions

We herein report that metalloporphyrin aggregation under homogeneous electrocatalysis conditions is a significant but previously unrecognized phenomenon that is generalizable across porphyrin substitution patterns and metal identity. Considering electrochemical CO_2_ reduction as a case study, we report a series of iron porphyrin catalysts bearing two, three, or four *meso* phenyl substituents to subtly tune dispersive interactions that alter the propensity of these catalysts to aggregate. Aggregation under electrochemically relevant conditions was confirmed with DLS and UV–vis spectra of the porphyrin Soret and Q bands, where spectral red-shifts support the formation of J-aggregate (staircase) structures in solution. Inverse relationships between catalyst concentration and activity indicate that metalloporphyrin aggregation inhibits catalytic performance and that this effect becomes more severe as the number of *meso* phenyl groups increases. Concentration-dependent electrochemical behavior shows that the number of *redox-active* species increases linearly with catalyst concentration, while the number of *catalytically active* species decreases. These findings are consistent with the formation of solution aggregates that maintain electronic conductivity to every porphyrin in the stack, but where buried porphyrins are not catalytically active. These spectroscopic and CV observations translate to bulk electrocatalytic performance, where up to 75% of dissolved catalyst is inactive at 1 mM compared to at 0.25 mM. We further demonstrate that porphyrin aggregation state can be perturbed by titration of certain additives and modification of ligand structures, two simple strategies that hold promise for tuning catalytic performance. Overall, it is imperative that catalytic parameters report on intrinsic activity rather than on underlying side phenomena or inhibition processes. This work highlights simple electrochemical and spectroscopic experiments that can be used to identify the presence and catalytic consequences of metalloporphyrin aggregation effects, ultimately leading to more accurate catalyst performance evaluations.

## Supporting Information Available

Synthetic procedures and characterization, details of experimental procedures, supplemental electrochemical, spectroscopic, and computational data (PDF)
Structure files of optimized porphyrin aggregates (ZIP)
