# Supplementary material for: Porphyrin Aggregation under Homogeneous Conditions Inhibits Electrocatalysis: A Case Study on CO2 Reduction
Source: ACS Cent Sci. 2024 Jun 3;10(6):1251–61. doi: 10.1021/acscentsci.4c00121 (PMC11212130; doi:10.1021/acscentsci.4c00121)
Supplement: Supplementary file 1 — oc4c00121_si_001.pdf [file oc4c00121_si_001.pdf]

Supplementary Information

Porphyrin Aggregation under Homogeneous Conditions Inhibits Electrocatalysis: A Case Study on CO_2 Reduction

Kaitlin L. Branch[†], Erin R. Johnson[‡] and Eva M. Nichols[*,†]

[†]Department of Chemistry, The University of British Columbia, 2036 Main Mall, Vancouver, British Columbia, V6T 1Z1, Canada

[‡] Department of Chemistry, Dalhousie University, 6274 Coburg Rd, Halifax, Nova Scotia, B3H 4R2, Canada

* Email: enichols@chem.ubc.ca

Contents

S1. General Methods........ 3
S2. Synthetic Procedures........ 3
S3. General Electrochemistry Methods 4
S4. Brief Discussion of Relevant Previous Reports 6
S5. Details of Foot-of-the-Wave Analysis (FOWA) and k_{obs} Calculations 6
S6. Details of UV-Vis Aggregation Studies 7
S6.1 General Methods 7
S6.2 Concentration-Normalized Soret Band Shifts 8
S7. Scan Rate Dependence Experiments........ 8
S8. Rate Law Investigations Details and Data........ 11
S9. Representative FOWA Plots........ 16
S10. Summary of Controlled Potential Electrolysis Results 19
S10.1 Tabulated Faradaic Efficiencies 19
S10.2 Post-CPE Catalyst Characterization 19
S11. Details of Titration Experiments........ 20
S11.1 Pyrene Titration Experiments 20
S11.2 Chloride Abstraction Titration Experiments 22
S12. Spectroelectrochemical Aggregation Studies........ 24
S13. Details of Computational Modeling of Porphyrin Aggregates........ 26
S14. Survey of Additional Metalloporphyrin Aggregation 27
S14.1 Survey of Substituted Iron Tetraphenylporphyrins........ 27
S14.2 Survey of Various Metallo-Tetraphenylporphyrins 28
S15. Spectra and Characterization 30
References........ 32

S1. General Methods

Reagents and solvents were purchased from Sigma-Aldrich or Oakwood Chemical and used without further purification unless otherwise noted. The free-base porphyrin ligands *meso*-5,15-diphenylporphyrin (H_2DiPP) and *meso*-tetraphenylporphyrin (H_2TetraPP) were purchased from Frontier Scientific. THF was dried using a Pure Process Technology (Nashua, NH) solvent purification system. ^{1}H NMR spectra were recorded on Bruker Avance 300 MHz spectrometer. Chemical shifts were referenced to residual proteo-solvent signal. Electrospray ionization mass spectra (ESI-MS) were collected on a Micromass LCT time-of-flight instrument in LC-MS grade methanol. UV-Vis spectra were recorded using an Agilent Cary 60 spectrophotometer at room temperature with a 10 mm micro rectangular quartz glass cuvette for characterization and a short-path 1 mm SEC-CT thin layer quartz glass cuvette (AirekaCells) for aggregation studies and spectroelectrochemical experiments. Dynamic light scattering (DLS) experiments were performed on a NanoBrook Omni particle size analyzer (Brookhaven) with an incident light of 640 nm in a 10 mm quartz cuvette at 19.5°C. Samples were prepared in DMF electrolyte (0.1 M $TBAPF_6$) and filtered through a 0.45 μm membrane filter. The refractive index of this solvent was measured to be 1.4306 at 19.5°C with an Abbe Refractometer (Atago). Anhydrous DMF for electrochemical experiments was purchased from Sigma-Aldrich and stored in an amber glass bottle over molecular sieves in a nitrogen-filled glovebox. Tetrabutylammonium hexafluorophosphate ($TBAPF_6$) supporting electrolyte was purified *via* three subsequent recrystallizations from ethanol and stored in a sealed desiccator. High purity gas cylinders (CO_2, Ar) were purchased from Linde. No unexpected or unusually high safety hazards were encountered.

S2. Synthetic Procedures

Preparation of *meso*-5,10,15-triphenylporphyrin (TriPP)

Synthesis of TriPP was performed according to a modified literature procedure.[1] Free-base *meso*-5,15-diphenylporphyrin (100 mg, 0.22 mmol, 1 eq) was added to an oven-dried Schlenk flask containing a Teflon stir-bar under nitrogen atmosphere. The flask was charged with dry THF (70 mL) and cooled in a dry-ice acetone bath (−78 °C), after which a solution of phenyllithium (1.9 M in dibutyl ether, 1.15 mL, 10 eq) was added dropwise *via* syringe. The solution was stirred at −78 °C for 2 hours, after which the solution was removed from the cold bath and stirred for an additional 1.5 hours. Over the course of the reaction at room temperature, the solution changed from deep purple to green-brown in colour. The mixture was quenched with a 50:50 mixture of H_2O and THF (30 mL), and stirred for 15 minutes. Subsequently, 2,3-dichloro-5,6-dicyano-1,4-benzoquinone (DDQ) (200 mg, 0.88 mmol, 4 eq) was added with another 15 minutes of stirring, then the solvent was removed under vacuum. Final purification was achieved *via* column chromatography (silica gel, 8:1 hexane:ethyl acetate) to afford the pure final product (84 mg, 71 % yield).

TriPP. Purple solid. **^{1}H NMR (300 MHz, $CDCl_3$)** δ (ppm) 10.22 (s, 1H, *meso*-H), 9.35 (d, J = 4.6 Hz, 2H, β-H), 9.03 (d, J = 4.6 Hz, 2H, β-H), 8.89 (q, J = 4.8 Hz, 4H, β-H), 8.24 (td, J = 8.5, 7.0, 4.0 Hz, 6H, *o*-Ph-H), 7.86 – 7.68 (m, 9H. *m,p*-Ph-H), -2.98 (s, 2H, NH). **MS (ESI)** observed (m/z): 539.2; calculated for $[M+H]^+$ $C_{38}H_{27}N_4$: 539.22. **UV-Vis** (MeOH) λ_{max} (nm) 408, 507, 541, 583, 637.

Metallation Procedures

A solution of free-base porphyrin (1 eq) in minimal DMF (~5 mL) was added to a solution of $FeCl_3 \cdot 6H_2O$ (12 eq) in a minimal amount of DMF (~1 mL). The resulting solution was stirred at reflux for about 3 hours under nitrogen. The reaction completion was confirmed *via* UV-Vis, at which point the cooled solution was neutralized with HCl (6 M, 10 mL). The resulting precipitate was collected *via* vacuum filtration and washed with HCl (3 M, 10 mL) and excess water. The precipitate was dried under reduced pressure overnight to afford the final complex. Metalations were performed on scales between 0.05-0.10 mmol of free-base porphyrin.

FeDiPP. Brown Solid. Yield: 91 % **MS (ESI)** observed (m/z): 516.0; calculated for $[M]^+$ $C_{32}H_{20}FeN_4$: 516.10. **UV-Vis** (DMF) λ_{max} (nm) 401, 561, 657.

FeTriPP. Brown Solid. Yield: 90 % **MS (ESI)** observed (m/z): 592.0; calculated for $[M]^+$ $C_{38}H_{24}FeN_4$: 592.14. **UV-Vis** (DMF) λ_{max} (nm) 407, 566, 610, 671.

FeTetraPP. Brown Solid. Yield: 87 % **MS (ESI)** observed (m/z): 668.1; calculated for $[M]^+$ $C_{44}H_{28}FeN_4$: 668.17. **UV-Vis** (DMF) λ_{max} (nm) 414, 569, 616, 680.

S3. General Electrochemistry Methods

Cyclic Voltammetry Details

Cyclic voltammograms (CVs) were performed in dry DMF containing 0.1 M $TBAPF_6$ supporting electrolyte under Ar, CO_2, or Ar/CO_2 mixtures. CVs were performed with a SP-50 potentiostat (Bio-Logic) using a three-electrode set-up: a 3.0 mm diameter glassy carbon working electrode (Bioanalytical Systems, Inc.), a platinum wire counter electrode (0.5 mm diameter), and a silver wire encased in a Vycor tip glass tube filled with 0.1 M $TBAPF_6$ electrolyte as the pseudo-reference electrode. The working electrode was polished between each scan with a slurry of water and alumina (0.05 µm) on a felt pad, then rinsed with water followed by acetone and dried with a stream of pressurized air. An initial blank scan of the electrolyte solution was performed before each experiment. Following the experiment, the pseudo-reference electrode was referenced to the ferrocene/ferrocenium (Fc/Fc^+) redox couple. All CVs were compensated for internal resistance at 85% compensation of the uncompensated resistance (R_u).

Controlled Potential Electrolysis Details

Controlled potential electrolysis (CPE) experiments were performed using a CHI650E potentiostat (CH Instruments, Inc.) in a gas-tight custom-made PEEK cell similar to those previously reported.[2] The cell consists of two compartments separated by a glass frit. The working compartment houses a glassy carbon working electrode (1 cm^2) and a silver wire encased in a Vycor tip glass tube filled with 0.1 M $TBAPF_6$ electrolyte as the pseudo-reference electrode. The counter compartment houses a graphite rod counter electrode (surface area ≈ 8 cm^2) . The solvent (DMF) was pre-sparged with argon for 20 minutes before use to remove any residual dimethylamine impurity. The working compartment was prepared with the desired concentration of catalyst and 100 mM PhOH in 7 mL 0.1 M $TBAPF_6$ in DMF. The counter compartment was prepared with 3 mL electrolyte solution (either 0.1 M $TBAPF_6$ in DMF, or 0.1 M TBAOAc in DMF as a sacrificial substrate). The cell was then sparged for 30 minutes with a mixture of 95% CO_2 and 5% He (internal standard), prepared with precision mass flow controllers (Alicat Scientific). The electrolysis was performed at a potential −0.3 V from the onset of the catalytic wave (~ −2.2 V vs. Fc/Fc^+), where the

exact potential was determined prior to each experiment by running a CV in the cell before electrolysis and aligning based on the observed catalytic onset potential. Following a 90-minute electrolysis, the headspace of the cell was directly injected into the pre-evacuated sample loops (−20 bar passive vacuum) of a gas chromatograph (SRI Multiple Gas Analyzer #5) through a Quick-Connect valve (Swagelok). An in-line thermal conductivity detector (TCD) was used to detect He and H_2, while a flame ionization detector (FID) with a methanizer was used to detect CO (**Figure S1**). The amount of gaseous products produced was determined by comparing the ratio of product gas to internal standard peak integrals with a prepared calibration curve (**Figure S2**).

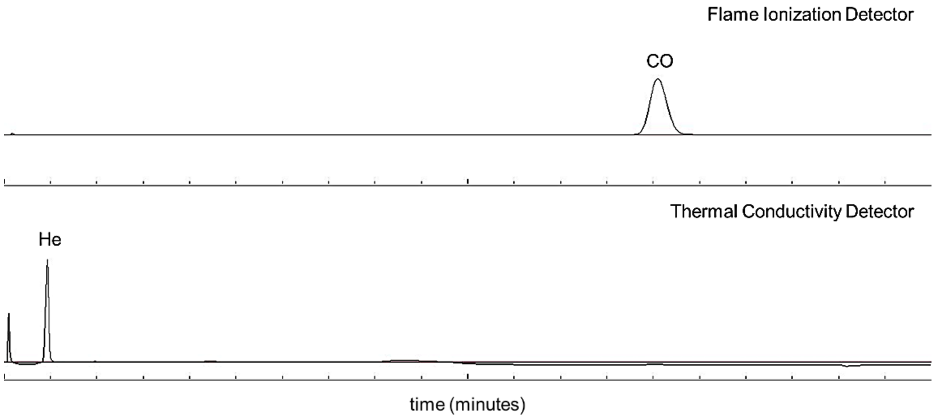

Figure S1. Representative GC trace of post-electrolysis sample headspace. Top: FID detector showing CO peak. Bottom: TCD detector showing He internal standard peak. Time axis demarcations represent 1 minute intervals.

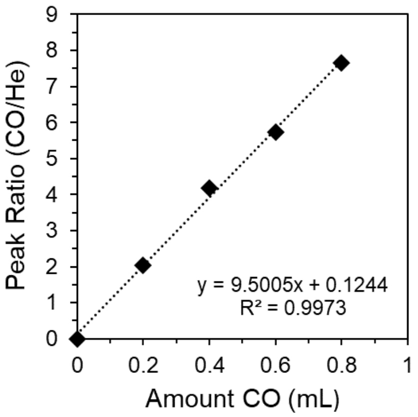

Figure S2. GC calibration curve for CO product formation with He as the internal standard.

S4. Brief Discussion of Relevant Previous Reports

Literature examples of investigations into the catalyst concentration dependence term in the rate law for metalloporphyrin electrocatalysts are limited, as most often a first-order assumption is made without experimental verification. As a result, there has been very limited discussion in the literature on the possible role of catalyst concentration—and porphyrin aggregation—on performance.

A small number of previous reports have compared metalloporphyrin catalytic currents[3,4] or rate constants[2,5–7] measured at variable catalyst concentrations to gain insight into this term in the rate law, and several have demonstrated agreement with a first-order catalyst concentration dependence. Conversely, one example observed a notable inverse relationship between the concentration of iron porphyrin catalyst and activity which was attributed to solution dimerization, however this hypothesis was not further explored or discussed in detail.[6] Together, these examples provide precedent for both agreement and contradiction with a first-order assumption in catalyst; however, since many of these examples feature elaborate catalyst designs (i.e., with appended pendant groups) and variable operating conditions (i.e., solvent, electrolyte, or additives), they are unable to provide an extensive understanding of catalyst concentration dependence in the rate law or the role of catalyst aggregation.

S5. Details of Foot-of-the-Wave Analysis (FOWA) and k_{obs} Calculations

The catalytic rate law for the electrocatalytic reduction of CO_2 can be described as follows:

$$\text{Rate} = -\frac{\text{dCO}_2}{\text{dt}} = k_{\text{obs}}[\text{catalyst}]^{\text{z}} = k_{\text{cat}}[\text{acid}]^{\text{x}}[\text{CO}_2]^{\text{y}}[\text{catalyst}]^{\text{z}}$$

Where the subscripts x, y, and z are the reaction orders in exogenous acid, CO_2 substrate and catalyst, respectively. In the subsequent analysis procedure to calculate observed rate constants, z=1 is assumed.[8]

Observed rate constants (k_{obs}) for the electrocatalytic reduction of CO_2 were calculated from cyclic voltammograms using Foot-of-the-Wave Analysis (FOWA) as developed and described by Savéant and coworkers.[8] Assuming each catalyst presented in this work follows the same mechanism as previously reported,[8] the reduction of CO_2 with iron porphyrins can be described as an EC' process:

$$\text{Fe(I)}^- + \text{e}^- \rightleftharpoons \text{Fe(0)}^{2-}$$

$$\text{Fe(0)}^{2-} + \text{CO}_2 + 2\text{H}^+ \rightarrow \text{Fe(II)CO} + \text{H}_2\text{O}$$

$$\text{Fe(II)CO} + \text{e}^- \rightarrow \text{Fe(I)}^- + \text{CO}$$

The rate-determining step (RDS) includes pre-equilibrium CO_2 binding to the Fe(0) active site followed by subsequent proton-coupled electron transfer. As a result, the following relationship can be derived:

$$\frac{i}{i_p^0} = \frac{2.24(n_{cat})^{\sigma}\sqrt{\frac{k_{obs}}{fv}}}{1 + \exp\left[f(E - E_{cat}^0)\right]}$$

Where *i* is current, i_p^0 is the peak current, E is the potential, *v* is the scan rate (in V/s), f= F/RT = 38.94 V^{-1}, and n^{σ} is a constant that describes the number of electrons required for catalysis (*n* = 2) to the power of a variable (σ) which describes the electron transfer process as being solely from the electrode (σ = 1), or resulting from disproportionation between iron species in solution (σ = 0.5). A value of σ = 1 is used as it has been proposed that this value will provide the most conservative estimate of k_{obs}.[9,10] That is, the

calculated rate constants when σ = 1 is used will not be overestimated under any of the mechanistic possibilities for electron transfer, as this equation will provide the lower-limit values of k_{obs}.

Prior to catalytic investigations, an initial CV collected under argon in the absence of PhOH was used to determine E^0_{cat}, the reduction potential of the $Fe^{I/0}$ couple, and the peak current (measured at the formal reduction of $Fe^{II/I}$) (i^0_p). In concentration-dependence experiments, i^0_p was determined individually for each concentration tested. From this, a "Foot-of-the-Wave" (FOW) plot can be constructed by plotting i/i^0_p against $(1 + \exp[f(E - E^0_{cat})])^{-1}$. Fitting of this plot to a linear function yields a line with slope $2.24(n_{cat})^{\sigma}\sqrt{k_{obs}/fv}$, from which k_{obs} can be calculated. To ensure consistency between k_{obs} values determined within this study, all linear functions were fit up to an x-axis value of 0.1, or until an R^2 of 0.98 was achieved.

We additionally note that since the equations used in FOWA assume a first-order dependence on catalyst concentration,[8] FOWA cannot be used to *explicitly* extract the order in catalyst concentration but can only highlight deviations from the implicitly assumed result. When this assumption is not supported experimentally, the rate constants derived from FOWA are not directly reporting inherent catalytic activity. Nevertheless, the observed rate constants extracted from FOWA can still inform agreement or disagreement with the first-order assumption and can be used to investigate trends in catalytic activity.

S6. Details of UV-Vis Aggregation Studies

S6.1 General Methods

UV-Vis aggregation studies were conducted in DMF containing 0.1 M $TBAPF_6$ electrolyte in a short path (1 mm) cuvette at room temperature. Solutions were prepared first at the largest concentration, then were sequentially diluted to survey the same concentration range as in the catalytic concentration-dependence studies (as indicated; 2.0, 1.5, 1.0, 0.5, 0.25, 0.125 mM). For investigations into the Soret Band, more dilute concentrations were used (as indicated; 0.1 mM – 0.0075 mM). The cuvette containing porphyrin solution was sonicated for 2 minutes prior to each measurement, and a scan was recorded at t = 0 min and at t = 20 min; no differences were observed between the spectra taken at these two time points. To normalize each spectrum to concentration, the absorbance values were divided by the concentration of porphyrin in solution, and the resulting concentration-normalized spectra were overlayed.

Previous studies have described µ-oxo dimer formation of **FeTetraPP** at low concentrations in DMF due to residual water.[11] In order to evaluate the potential contribution of µ-oxo dimer formation in our system, the features in our porphyrin concentration-dependent spectra were compared to those of a µ-oxo dimer prepared *via* titration with TBAOH (Figure S3). At higher concentration regimes (Figure S3a), the low intensity porphyrin Q bands (560, 610 nm) are similar to those of the µ-oxo dimer. This comparison reveals either a small amount of µ-oxo dimer formation and/or a coincidental similarity between features associated with µ-oxo dimerization and aggregation. Dynamic light scattering (DLS) data (Figure 2b) unequivocally show that aggregates of increasing size are present at higher concentrations, thereby indicating that the UV-Vis changes correlate with aggregation at least in part. Additionally, the spectral features observed upon porphyrin dilution (Figure 2c) are also seen upon titration with pyrene as a disaggregating agent (Figure 5a) that we speculate is not capable of breaking apart covalently bound µ-oxo dimers. Quantification of the relative amounts of aggregated metalloporphyrins vs. µ-oxo dimers is

not possible due to the similar UV-Vis characteristics of these species and the inability of other analytical methods to report on the specific chemical composition.

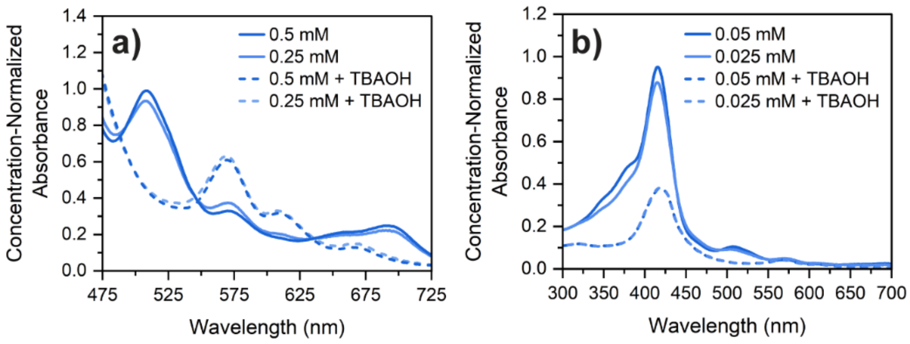

Figure S3. Comparison of variable concentration UV-Vis absorption spectra in (a) the Q band and (b) the Soret band regions for **FeTetraPP** (solid lines) and the μ-oxo dimer of **FeTetraPP** (dashed lines), formed *in situ* by addition of 5 equivalents of tetrabutylammonium hydroxide (TBAOH). Conditions: indicated catalyst concentration, 0.1 M $TBAPF_6$ in DMF, 1 mm path length.

S6.2 Concentration-Normalized Soret Band Shifts

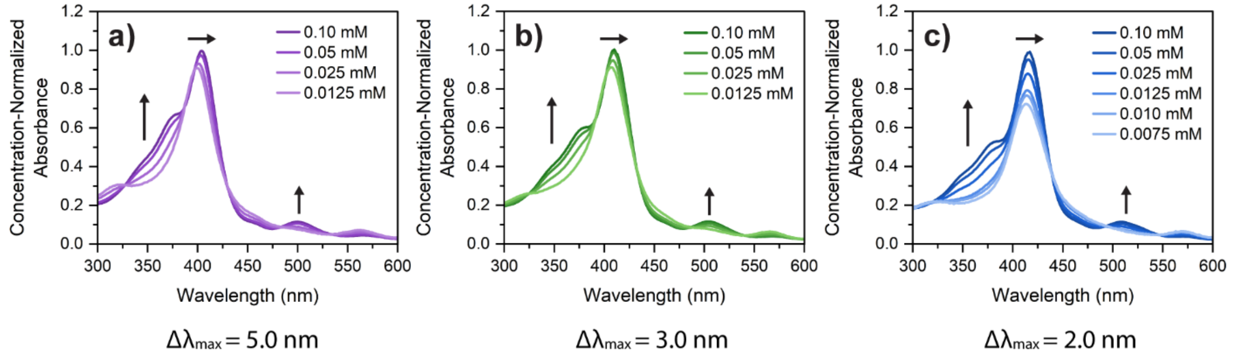

Figure S4. UV-Vis absorption spectra as a function of catalyst concentration under catalytically-relevant conditions at 0.1–0.0075 mM of (a) **FeDiPP** (b) **FeTriPP** and (c) **FeTetraPP** showing a red-shift of the Soret Band. Arrows are depicting spectral changes with increasing concentration. The magnitude of the Soret Band shift ($\Delta\lambda_{max}$) between the low and high concentration spectrum is reported below each respective plot. Conditions: indicated catalyst concentration, 0.1 M $TBAPF_6$ in DMF, 1 mm path length.

Note that changes in the shoulder peak of the Soret band (**Figure S4**) are generally understood to corelate with changes in axial ligation. Thus, these changes are likely related to equilibria between chloride-bound and solvent-bound iron centers as a function of dilution.

S7. Scan Rate Dependence Experiments

Scan rate dependence experiments were performed by preparing a solution of each iron porphyrin (2.0 mM) in 0.1 M $TBAPF_6$ in DMF. The solution was sparged thoroughly (15 min) with argon, then an initial scan was taken. Scans were then taken sequentially following dilutions of the CV solution with electrolyte

solution to analyze each of the catalyst concentrations (as indicated; 2.0, 1.5, 1.0, 0.5, 0.25, 0.125 mM). At each catalyst concentration, CVs were taken at several scan rates (as indicated; 50, 100, 250, 500, 750, 1000 mV/s). The solution was kept under an argon atmosphere throughout the experiment, and the working electrode was polished before each scan. Peak currents of each iron redox couple were measured at each scan rate and plotted against the square-root of scan rate, whereby according to the Randles–Ševčík equation:

$$i_p^0 = 0.446nFAC^0\left(\frac{nFvD_o}{RT}\right)^{1/2}$$

a linear correlation between peak current (i_p^0) and the square-root of scan rate ($v^{1/2}$) is characteristic of a diffusional process without the presence of electrode-bound species. A linear result was obtained for each catalyst and for each iron redox couple (**Figure S5**).

The slopes of each linear correlation in Figure S5 were used to calculate the diffusion coefficients (D_o) of each catalyst as a function of catalyst concentration (Figure S7). We hypothesized that an increase in porphyrin concentration and aggregate size would result in a decrease to the measured diffusion coefficients. When comparing the calculated diffusion coefficients, no clear trends are observed and the values do not significantly vary with catalyst concentration at any redox couple (Figure S7). We rationalize that diffusion coefficients may not be a reliable metric for aggregation severity because the expected change in D_o as a function of aggregate size is not well defined; examples in literature demonstrate that D_o can increase, decrease, or remain constant with increasing aggregate size.[12,13]

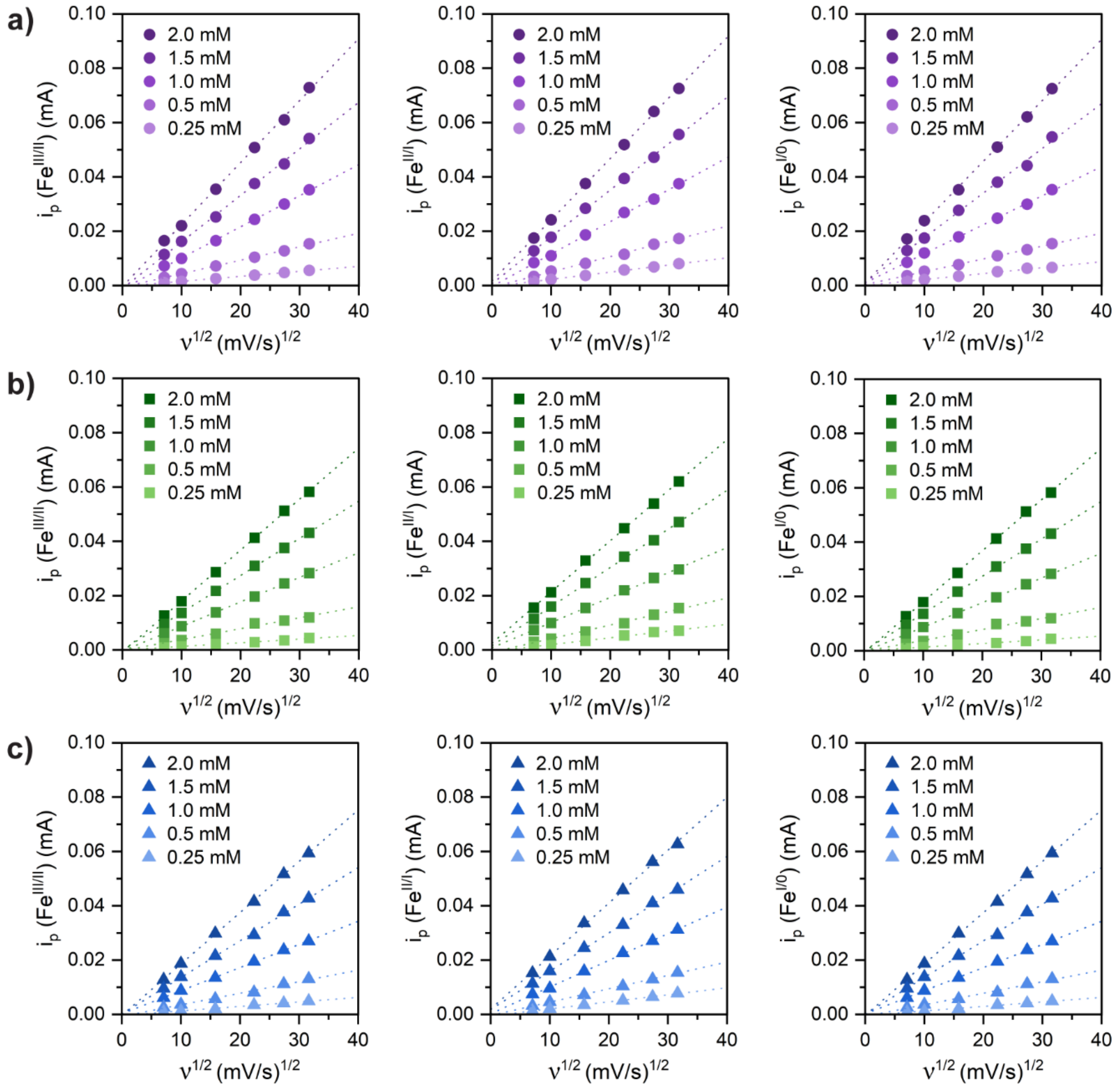

Figure S5. Scan rate dependence for (a) **FeDiPP** (b) **FeTriPP** and (c) **FeTetraPP**. Left: peak current of the $Fe^{III/II}$ redox couple. Middle: peak current of the $Fe^{II/I}$ redox couple. Right: peak current of the $Fe^{I/0}$ redox couple. Conditions: indicated catalyst concentration, 0.1 M $TBAPF_6$ in DMF under argon.

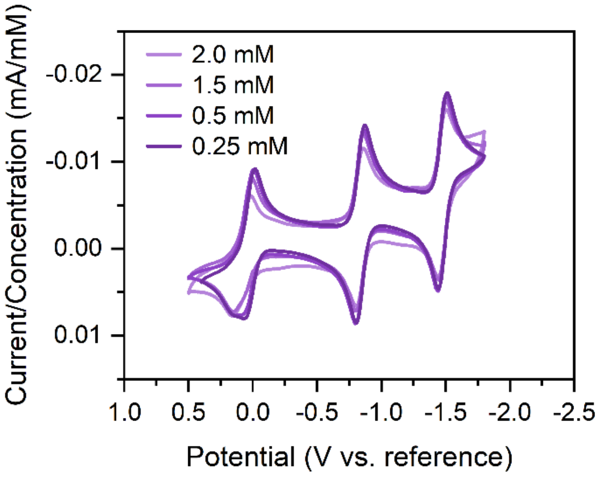

Figure S6. Representative CVs of **FeDiPP** at various concentrations. Conditions: indicated catalyst concentration, 0.1 M $TBAPF_6$ in DMF under argon, 100 mV/s.

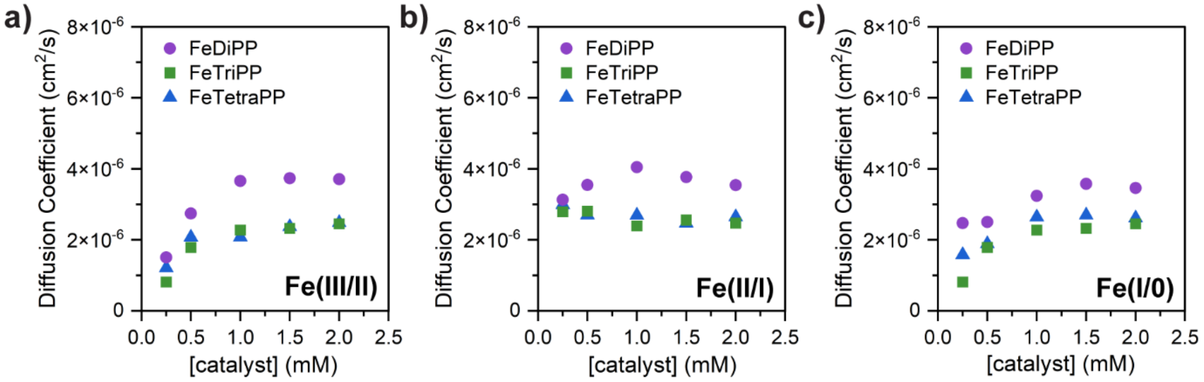

Figure S7. Diffusion coefficients calculated from scan rate dependence experiments according to the Randles–Ševčík equation using the peak current of (a) Fe(III/II) couple, (b) the Fe(II/I) couple, and (c) the Fe(I/0) couple. Conditions: indicated catalyst concentration, 0.1 M $TBAPF_6$ in DMF under argon.

S8. Rate Law Investigations Details and Data

Phenol Concentration Dependence Experiments

A solution of iron porphyrin (0.5 mM or 1.0 mM, as indicated) was prepared in 0.1 M $TBAPF_6$ in DMF. The solution was sparged thoroughly (15 min) with argon, then an initial scan was taken. A scan was then taken following saturation of the solution with CO_2. Scans were then taken sequentially following PhOH titrations (as indicated; 50, 100, 250, 500, 750, 1000 mM), whereby a scan under both argon and CO_2 were collected at each PhOH concentration. Solutions were sparged for 8 minutes between gases, and the working electrode was polished before each scan. The resulting CVs were analyzed by FOWA to calculate the observed rate constants (k_{obs}) at each PhOH concentration (**Figure S8**).

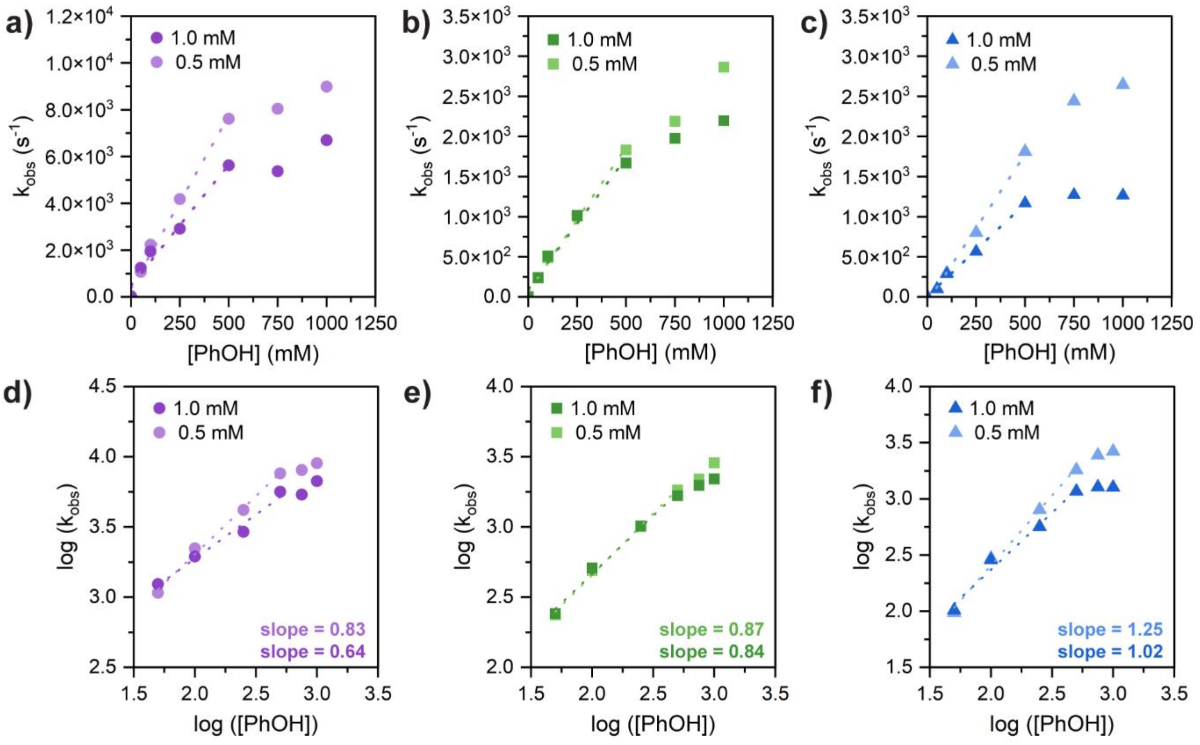

Figure S8. Observed rate constants (k_{obs}) as a function of PhOH concentration for (a) **FeDiPP** (b) **FeTriPP** and (c) **FeTetraPP** at 1.0 mM and 0.5 mM. Corresponding log/log plots for (d) **FeDiPP** (e) **FeTriPP** and (f) **FeTetraPP**; the linear fit and slopes include only the linear regime (the first 4 points). Conditions: Indicated PhOH and catalyst concentrations, 0.1 M $TBAPF_6$ in DMF, 100 mV/s scan rate.

Catalyst Concentration Dependence Experiments

A solution of iron porphyrin (2.0 mM) was prepared in 0.1M $TBAPF_6$ in DMF. The solution was sparged thoroughly (15 min) with argon, then an initial scan was taken. PhOH (250 mM) was then introduced to the solution, and both an argon and CO_2 scan were taken. Scans were then taken sequentially following dilutions of the CV solution (with a solution of 0.1 M $TBAPF_6$ in DMF containing 250 mM PhOH) to each of the catalyst concentrations (as indicated; 2.0, 1.5, 1.0, 0.5, 0.25, 0.125 mM). A scan under both argon and CO_2 were collected at each catalyst concentration. Solutions were sparged for 8 minutes between gases, and the working electrode was polished before each scan. The resulting CVs were analyzed by FOWA to calculate the observed rate constants (k_{obs}) at each catalyst concentration (**Table S1**), where the peak current (i_p^0) was measured individually for each catalyst concentration at the $Fe^{II/I}$ couple (**Figure S10**). We note that the peak currents are equivalent regardless of if the solutions are saturated with Ar or CO_2.

Table S1. Mean observed rate constant (k_{obs}) values and standard deviations as a function of iron porphyrin catalyst concentration, based on three independent CV measurements for each catalyst. Data is plotted in Figure 3c in the main text.

	FeDiPP		FeTriPP		FeTetraPP	
Catalyst Concentration (mM)	Mean k_{obs} (s^{-1})	Standard Deviation	Mean k_{obs} (s^{-1})	Standard Deviation	Mean k_{obs} (s^{-1})	Standard Deviation
2.00	600	± 110	420	± 130	310	± 74
1.50	1130	± 155	670	± 220	550	± 320
1.00	2100	± 268	1060	± 420	840	± 550
0.50	5060	± 187	2180	± 1360	1630	± 1240
0.25	9430	± 1250	3680	± 2640	3310	± 2890
0.125	19300	± 4290	4850	± 2970	4530	± 3470

The order in catalyst is inverse but cannot be fit by a simple linear function across the entire range of concentrations investigated (**Figure S9**). That is, the order in catalyst appears to be a function of catalyst concentration as a result of the changing aggregation state influencing the order in catalyst. In general, the tangent to log(k_{obs}) gets increasingly negative at higher catalyst concentrations (that is, the order in catalyst concentration gets more negative as catalyst concentration is increased). This is in agreement with a greater extent of aggregation and inhibition at higher catalyst loadings.

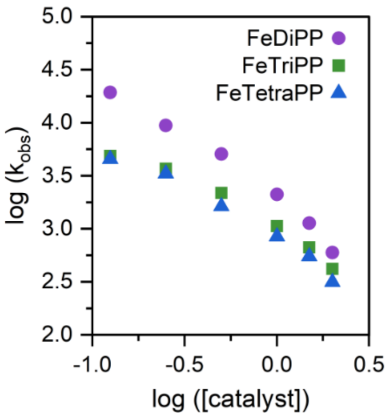

Figure S9. Log/log plots for catalyst concentration dependence experiments, showing a negative order in catalyst. Conditions: indicated catalyst concentration, 250 mM PhOH, 0.1 M $TBAPF_6$ in CO_2-saturated DMF, 100 mV/s scan rate.

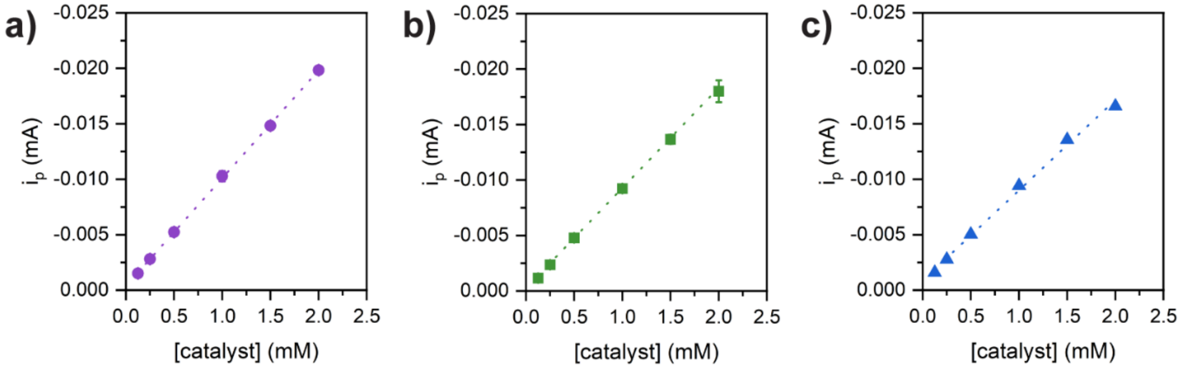

Figure S10. Average of $Fe^{II/I}$ peak current (i_p^0) values vs. catalyst concentration for (a) **FeDiPP** (b) **FeTriPP** and (c) **FeTetraPP**, showing a linear increase in peak current with increasing concentration as expected based on the Randles-Ševčík equation. Average i_p^0 from triplicate experiments, error bars represent 1 standard deviation; error bars not shown are smaller than their respective data marker. Conditions: indicated catalyst concentration, 250 mM PhOH, 0.1 M $TBAPF_6$ in CO_2-saturated DMF, 100 mV/s scan rate.

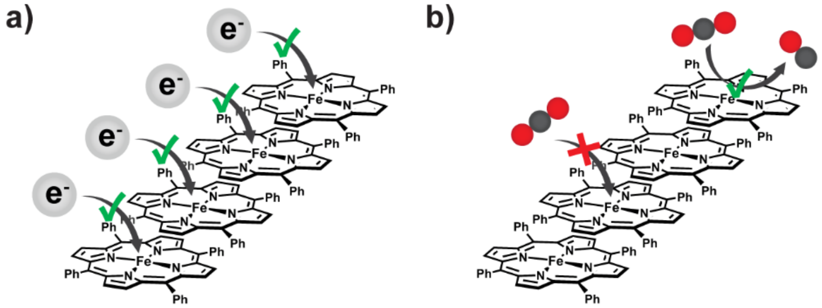

Figure S11. Schematic representation of electron transfer and catalysis in iron porphyrin aggregates (a) demonstrating electron transfer is possible within the porphyrin assembly, supported by a linear increase in peak current under argon with increasing catalyst concentration and (b) demonstrating the proposed catalytic inhibition process as a result of catalyst aggregation, whereby active sites within the assembly are likely inaccessible to substrate binding.

CO_2 Concentration Dependence Experiments

A solution of iron porphyrin (0.25 mM or 1.0 mM, as indicated) was prepared in 0.1 M $TBAPF_6$ in DMF. The solution was sparged thoroughly (15 min) with argon, then an initial scan was taken. PhOH (250 mM) was then introduced to the solution, and an additional argon scan was collected. Scans were then taken sequentially at different Ar/CO_2 mixtures (as indicated; 10, 20, 40, 50, 60, 80, 100 % CO_2) prepared with precision mass-flow controllers (Alicat Scientific). Solutions were sparged with each prepared gas mixture for 15 minutes, and the working electrode was polished before each scan. The resulting CVs were analyzed by FOWA to calculate the observed rate constants (k_{obs}) at each CO_2 concentration. The concentration of CO_2 in solution was assumed to be equal to the percent of CO_2 in the sparging mixture multiplied by the concentration in a CO_2-saturated solution of DMF (0.23 M).

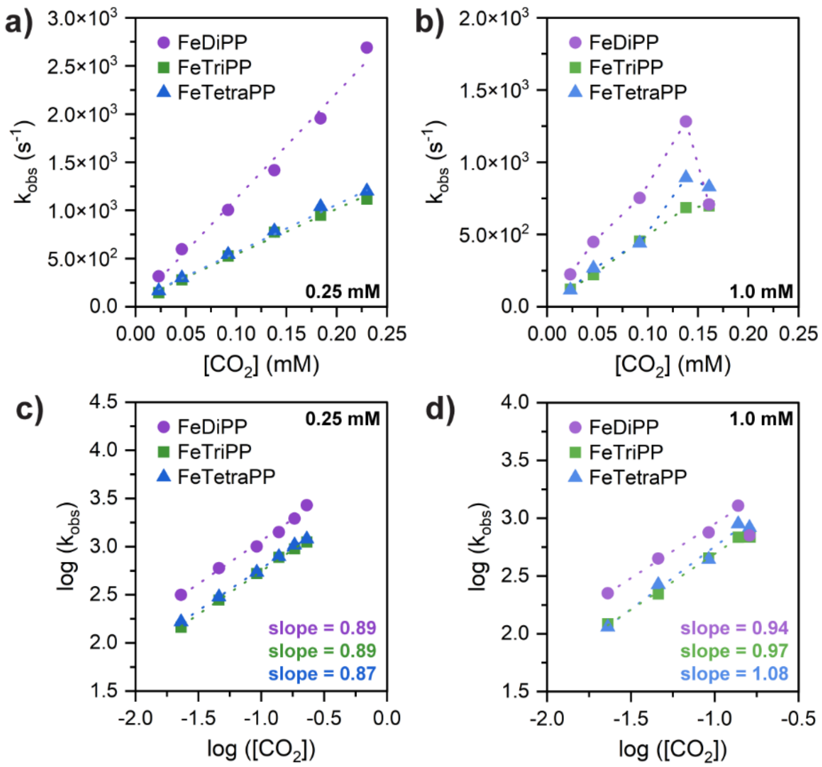

Figure S12. Observed rate constants (k_{obs}) as a function of CO_2 concentration at (a) 0.25 mM and (b) 1.0 mM catalyst. Corresponding log/log plots at (c) 0.25 mM and (d) 1.0 mM catalyst concentrations; the linear fit and slope in (d) include only the first 4 points. Conditions: 250 mM PhOH, indicated CO_2 concentration, 0.1 M $TBAPF_6$ in DMF, 100 mV/s scan rate.

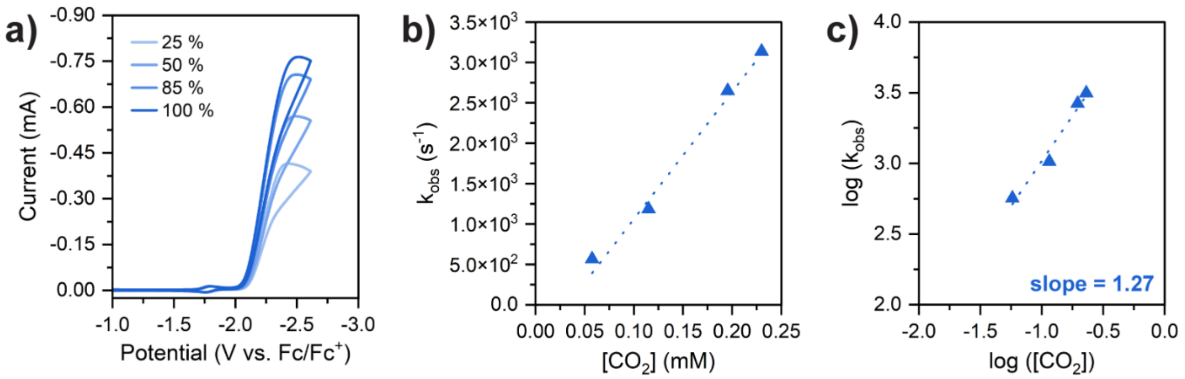

Figure S13. CO_2 dependence experiment at 500 mM PhOH. (a) CVs with increasing concentrations of CO_2 (b) observed rate constants (k_{obs}) as a function of CO_2 concentration (c) corresponding log/log plot showing a first order dependence in CO_2. Conditions: 1.0 mM **FeTetraPP**, 500 mM PhOH, indicated CO_2 concentration, 0.1 M $TBAPF_6$ in DMF, 100 mV/s scan rate.

S9. Representative FOWA Plots

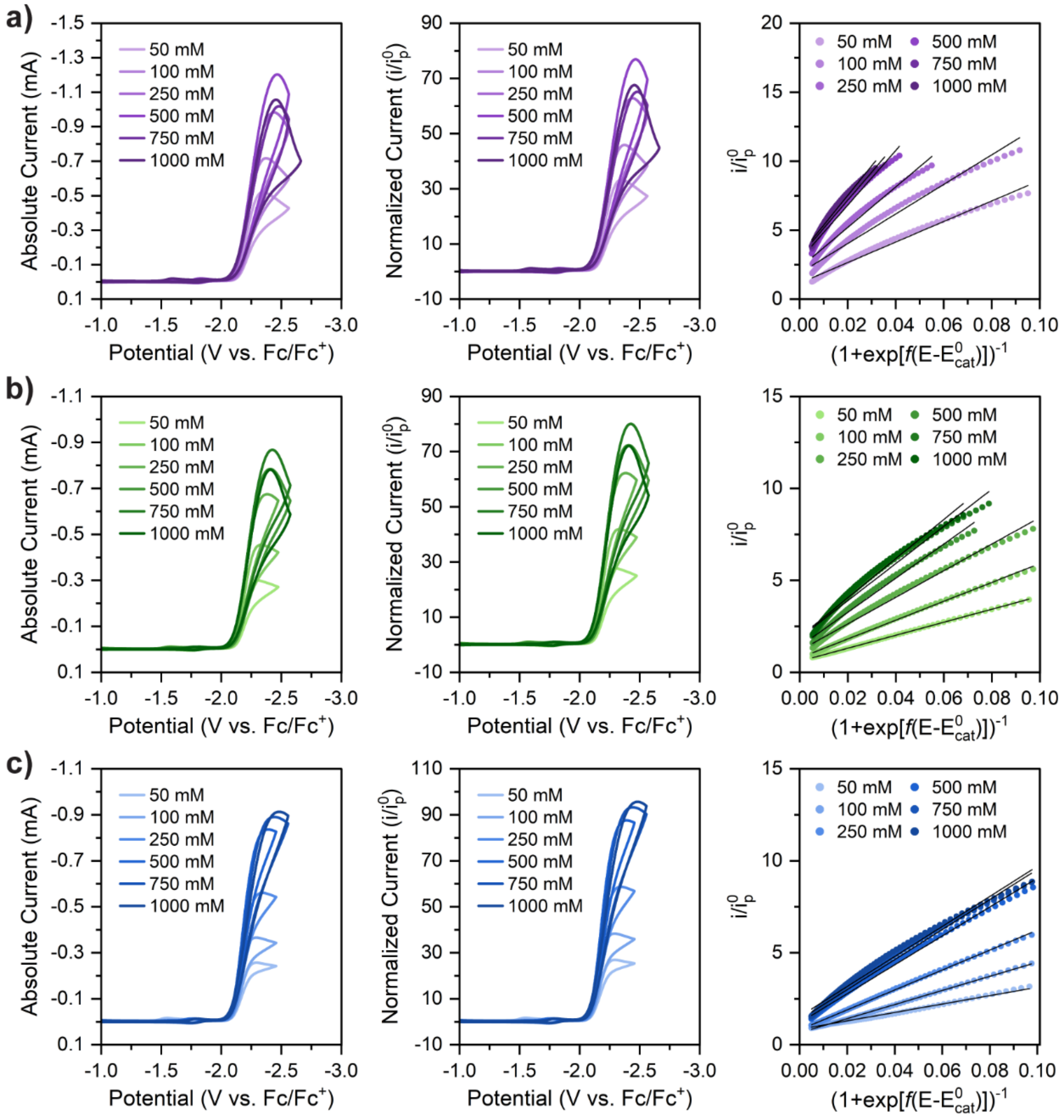

Figure S14. Representative PhOH concentration dependence FOWA for (a) **FeDiPP** (b) **FeTriPP** and (c) **FeTetraPP**. Left plots: cyclic voltammograms. Middle plots: normalized cyclic voltammograms (i/i_p^0). Right plots: FOW plots; black lines represent the fit used to extract k_{obs} values. Catalyst concentration is 1.0 mM; PhOH concentrations as indicated, 0.1 M $TBAPF_6$ in DMF, 100 mV/s scan rate.

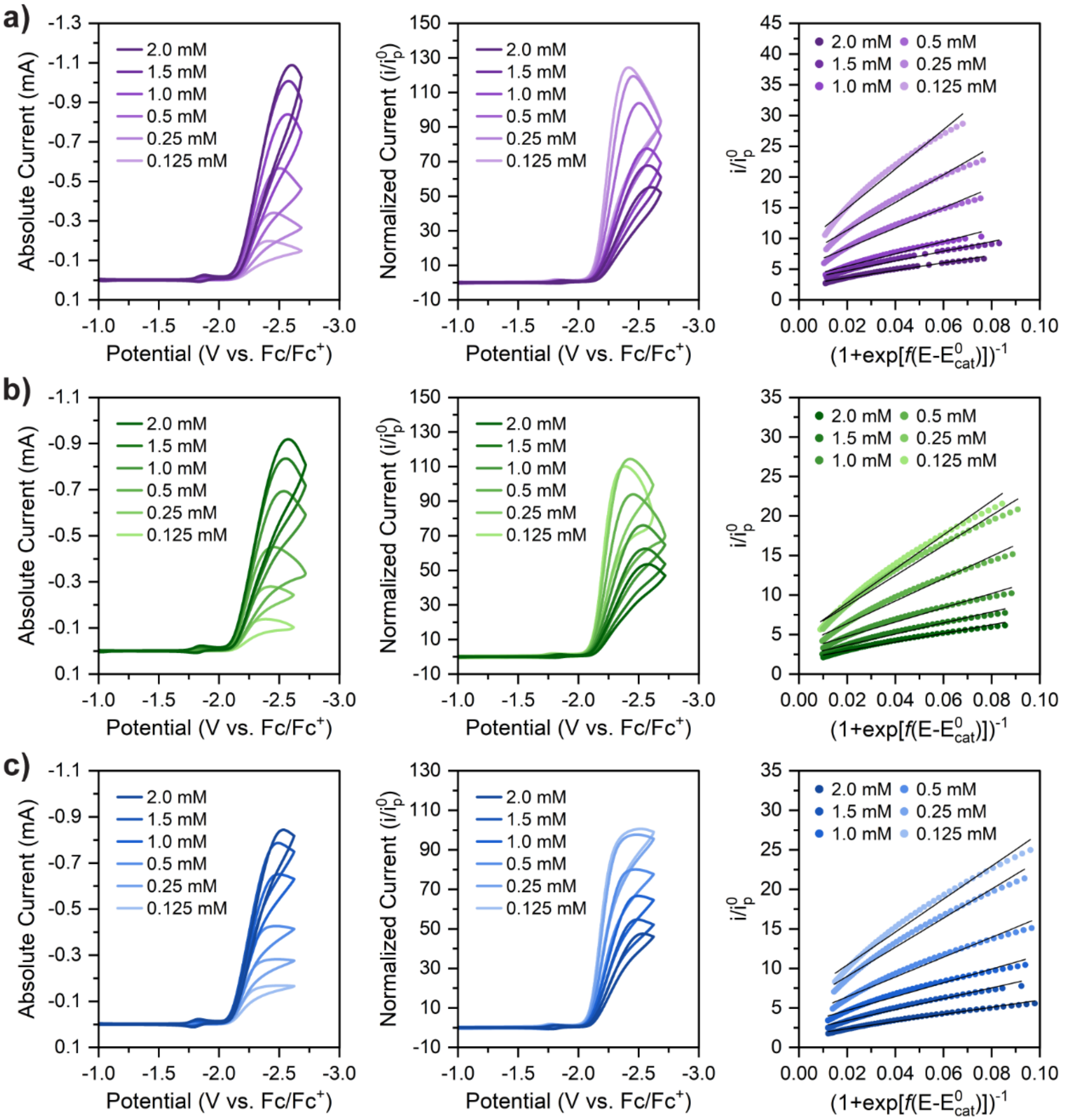

Figure S15. Representative catalyst concentration dependence FOWA for (a) **FeDiPP** (b) **FeTriPP** and (c) **FeTetraPP**. Left plots: cyclic voltammograms. Middle plots: normalized cyclic voltammograms (i/i_p^0). Right plots: FOWA plots; black lines represent the fit used to extract k_{obs} values. Catalyst concentrations as indicated, 0.1 M $TBAPF_6$ in DMF, 100 mV/s scan rate.

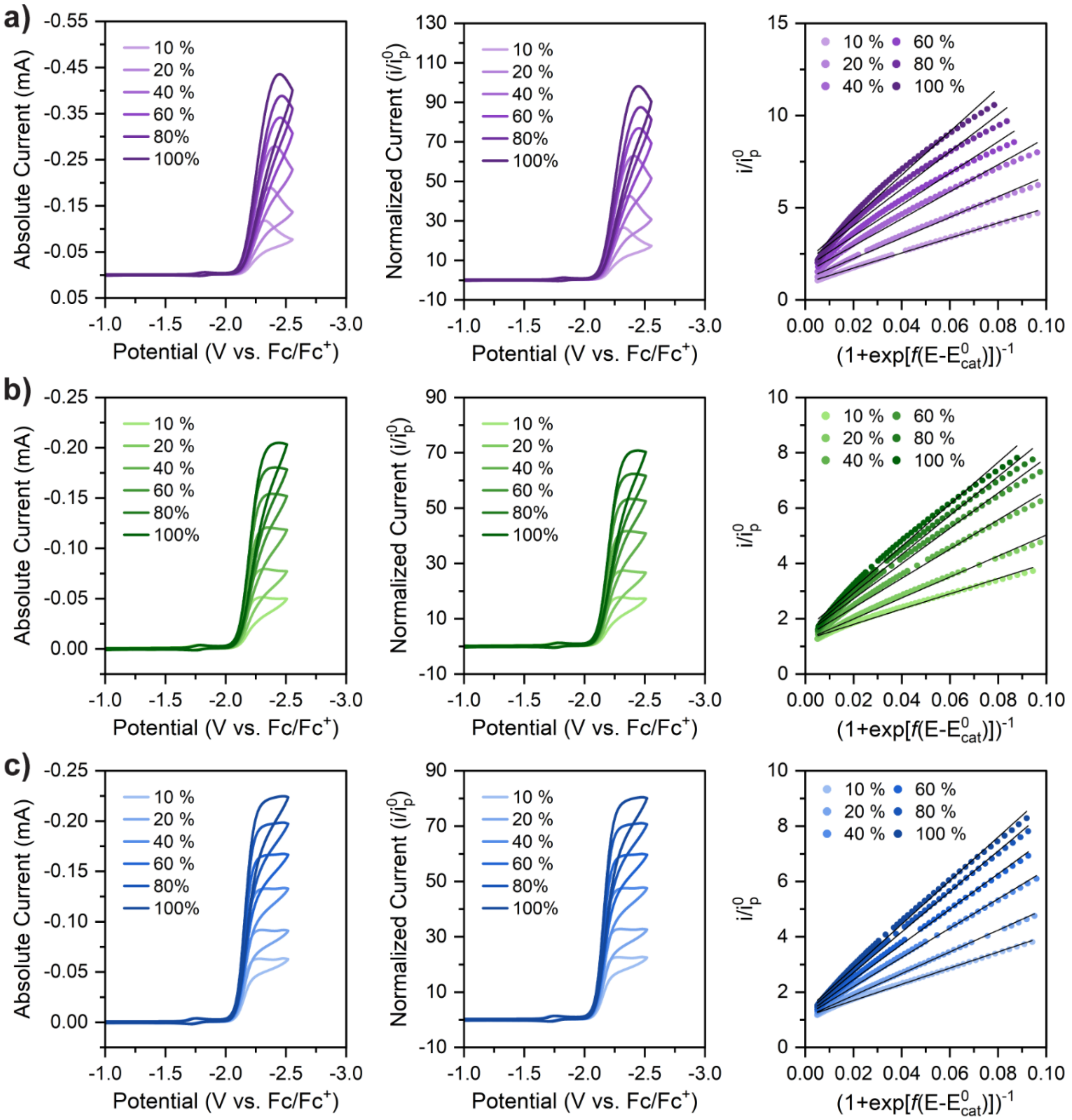

Figure S16. Representative CO_2 concentration dependence FOWA plots for (a) **FeDiPP** (b) **FeTriPP** and (c) **FeTetraPP**. Left plots: cyclic voltammograms. Middle plots: normalized cyclic voltammograms (i/i_p^0). Right plots: FOWA plots; black lines represent the fit used to extract k_{obs} values. Catalyst concentration is 0.25 mM, PhOH concentration is 250 mM, CO_2 concentration as indicated, 0.1 M $TBAPF_6$ in DMF, 100 mV/s scan rate.

S10. Summary of Controlled Potential Electrolysis Results

S10.1 Tabulated Faradaic Efficiencies

CPE experiments were performed in duplicate for each catalyst at both concentrations tested (0.25 mM and 1.0 mM). The average total charge passed and faradaic efficiencies are reported (**Table S2**). For each set of duplicate experiments, either 0.1 M $TBAPF_6$ electrolyte or 0.1 M TBAOAc (as a sacrificial substrate) electrolyte was used in the counter compartment to determine the amount of CO produced *via* solvent oxidation in the counter compartment. Experiments performed with TBAOAc as a sacrificial substrate in the counter compartment had on average a 9% lower FE for CO, suggesting some amount of solvent oxidation when $TBAPF_6$ is used.

Table S2. Results of CPE experiments, reporting charge and faradaic FE of CO for each CPE run.

Catalyst	Concentration	Charge (C)	Average Charge (± sdev)	FE (CO)	Average FE (± sdev)	TBAOAc
FeDiPP	1 mM	7.009	**7.42 ± 0.41**	99.7	**87.5 ± 12.2**	N
		7.838		75.3		Y
FeTriPP	1 mM	8.677	**8.51 ± 0.17**	87.5	**82.0 ± 5.6**	N
		8.340		76.4		Y
FeTetraPP	1 mM	7.190	**7.21 ± 0.02**	84.2	**77.2 ± 7.0**	N
		7.234		70.1		Y
FeDiPP	0.25 mM	6.317	**6.67 ± 0.35**	79.3	**80.3 ± 1.0**	N
		7.025		81.3		Y
FeTriPP	0.25 mM	4.553	**4.48 ± 0.07**	79.3	**76.7 ± 2.7**	N
		4.410		74.0		Y
FeTetraPP	0.25 mM	4.106	**4.45 ± 0.35**	81.1	**80.6 ± 0.5**	N
		4.796		80.0		Y

S10.2 Post-CPE Catalyst Characterization

Post-electrolysis solutions were studied to investigate potential catalyst decomposition. CV's were collected pre- and post-electrolysis directly in the CPE cell immediately before and after electrolysis. For all catalysts in the series and at both catalyst concentrations, there was no evidence of significant catalyst degradation as there were only minor decreases in the catalytic currents observed in CVs of post-electrolysis solutions (**Figure S17**). Post-CPE solutions were also analyzed *via* UV-Vis by removing a 0.1 mL aliquot from the working compartment solution and diluting into 1 mL DMF containing 0.1 M $TBAPF_6$ in a 1 mm path length quartz cuvette. Spectral measurements were taken at several time points to observe the re-oxidation of the iron porphyrin complexes following exposure to air. For each of the catalysts, the scans taken immediately upon sampling from the electrolysis cell show a sharp Soret band, characteristic of an Fe^{II} porphyrin species. Following this initial exposure to air, the iron is re-oxidized to the Fe^{III} species and the spectra agree with the pre-CPE spectra, showing no evidence of decomposition (**Figure S18**).

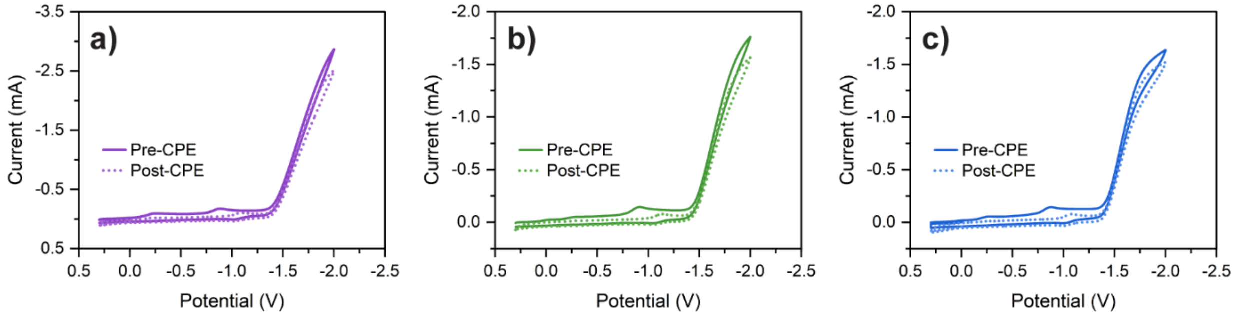

Figure S17. Representative CVs taken pre- and post-electrolysis (a) **FeDiPP** (b) **FeTriPP** (c) **FeTetraPP**. Conditions: 0.25 mM catalyst, 0.1 M $TBAPF_6$ in DMF, 100 mV/s scan rate.

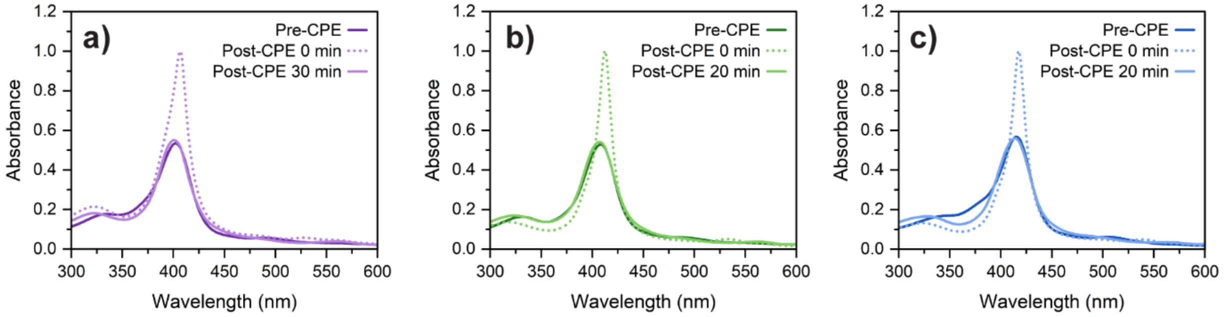

Figure S18. Representative UV-Vis absorption spectra taken pre- and post-electrolysis; (a) **FeDiPP** (b) **FeTriPP** (c) **FeTetraPP**. Conditions: 0.1 mM catalyst, 0.1 M $TBAPF_6$ in DMF, path length 1 mm.

S11. Details of Titration Experiments

S11.1 Pyrene Titration Experiments

<u>UV-Vis Pyrene Titration</u>

UV-Vis measurements were taken as described above (Section *S6.1*), except a constant concentration of **FeTetraPP** (1.0 mM) was used and spectra were recorded following titrations of a pyrene stock solution (40 mM) resulting in additions between 0 – 2 molar equivalents of pyrene. No pyrene absorptions appear within the porphyrin Q band region of interest.

<u>Electrochemical Pyrene Titration Experiment</u>

Prior to the pyrene titration experiment, CVs of pyrene were investigated to ensure no redox features would interfere with the CV analysis of **FeTetraPP** CO_2 reduction catalysis. CVs of pyrene (0.5 mM) were taken under argon and CO_2, then PhOH (10 mM) was added and again both an argon and CO_2 scan were taken (**Figure S19**). Pyrene shows some reduction events at around −2.5 V vs. Fc/Fc^+ under all conditions, thus the scan window for the subsequent pyrene titrations was cut off at this potential.

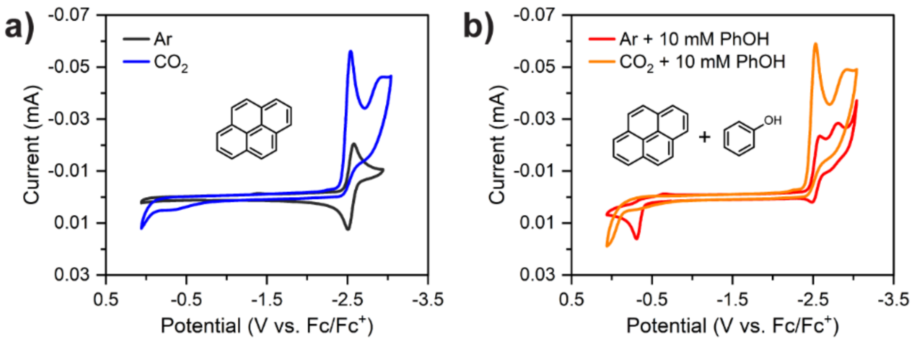

Figure S19. CVs of pyrene (0.5 mM) under argon and CO_2. (a) No PhOH (b) 10 mM PhOH. Conditions: indicated PhOH concentration, 0.1 M $TBAPF_6$ in DMF, 100 mV/s scan rate.

A solution of **FeTetraPP** (1.0 mM) was then prepared in 0.1 M $TBAPF_6$ in DMF. The solution was sparged thoroughly (15 min) with argon, then an initial scan was taken. PhOH (10 mM) was added to solution, and both an argon and CO_2 scan were taken. Scans were then taken sequentially following pyrene titrations (as indicated; 0.4, 0.8, 1.0, 1.4, 1.8, 2.0 mM) under CO_2. The solution was kept under a CO_2 atmosphere throughout the experiment, and the working electrode was polished before each scan.

Controlled Potential Electrolysis with Pyrene

A CPE with **FeTetraPP** (1.0 mM) and 1 molar equivalent of pyrene (1.0 mM) was performed to investigate how the presence of a disaggregating agent influences bulk CO_2 reduction performance (Figure S20). The CPE was performed as described above (Section *S3*) except with the addition of pyrene (1.0 mM) to the working compartment. The current and total charge passed were larger than that of 1.0 mM of **FeTetraPP** alone (8.34 C vs 7.21 C), consistent with disaggregation of the catalyst. However, the Faradaic efficiency for CO was slightly reduced (68.0 % FE vs 77.2 % FE), which we attribute to some amount of pyrene plating on the electrode surface and/or small amounts of pyrene reduction.

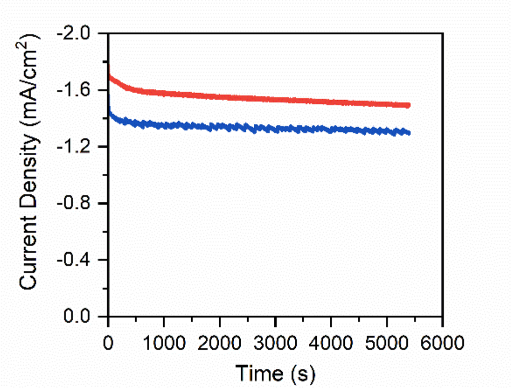

Figure S20. Controlled potential electrolysis experiments of 1.0 mM **FeTetraPP** (blue trace) and 1.0 mM **FeTetraPP** with 1.0 mM pyrene (red trace). Conditions: 100 mM PhOH, 0.1 M $TBAPF_6$ in CO_2-saturated DMF, 90 minutes at ~ −2.2 V vs. Fc/Fc^+.

UV-Vis PhOH Titration Experiment

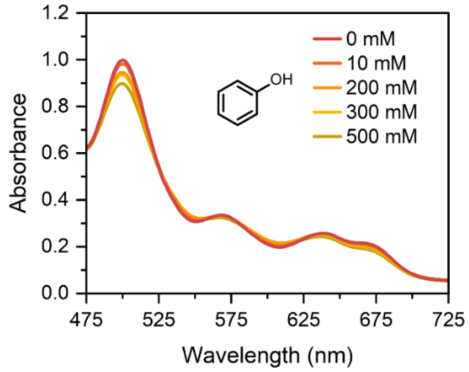

Figure S21. UV-Vis absorption spectra of the Q band region of **FeDiPP** upon titration of PhOH. Conditions: 1.0 mM **FeDiPP**, indicated PhOH concentration, 0.1 M $TBAPF_6$ in DMF, path length 1 mm.

S11.2 Chloride Abstraction Titration Experiments

UV-Vis Chloride Abstraction Titration

UV-Vis measurements were taken as described above (Section *S6.1*), except a constant concentration of **FeTriPP** (0.5 mM) was used and spectra were recorded following titrations of a silver hexafluorophosphate ($AgPF_6$) stock solution (4 mM) resulting in additions between 0 – 0.4 molar equivalents of $AgPF_6$.

Electrochemical Chloride Abstraction Titration

Prior to the chloride abstraction titration experiment, CVs of $AgPF_6$ were investigated to ensure no redox features would interfere with the CV analysis of **FeTriPP** CO_2 reduction catalysis. CVs of $AgPF_6$ (0.1 mM) were taken under argon at two different scan windows. A larger scan window (**Figure S22, black trace**) shows the redox features corresponding to silver. Thus, the scan window for the subsequent $AgPF_6$ titration was cut off at about -0.45 V vs. Fc/Fc^+ (**Figure S22, red trace**).

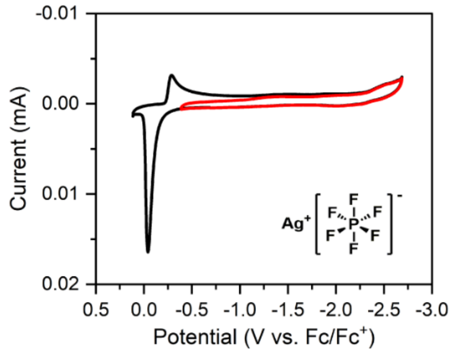

Figure S22. CVs of $AgPF_6$ (0.1 mM) blank under argon. Black trace: large scan window. Red trace: scan window used in titration experiment. Conditions: 0.1 M $TBAPF_6$ in DMF, 100 mV/s scan rate.

A solution of **FeTriPP** (0.5 mM) was then prepared in 0.1 M $TBAPF_6$ in DMF. The solution was sparged thoroughly (15 min) with argon, then an initial scan was taken. PhOH (10 mM) was added to solution, and both an argon and CO_2 scan were taken. Scans were then taken sequentially following titrations of $AgPF_6$ under CO_2. The solution was kept under a CO_2 atmosphere throughout the experiment, and the working electrode was polished before each scan.

Dilution Experiment in Conditions of Excess Chloride

To further understand the role of axial ligation on the aggregation of iron phenylporphyrins, we repeated the Soret band UV-Vis dilution study (**Figure S4**) in the presence of an excess of chloride ions (10 mM tetrabutylammonium chloride). Under these conditions, the disaggregation effect (that is, the red-shifting of the Soret band) previously observed upon dilution is no longer observed. This suggests that equilibrium chloride ligand exchange has a significant role in the aggregation state for these complexes; potentially, a greater amount of solvent-bound species is formed following dilution and this species is less prone to aggregation. Under conditions of excess chloride, the equilibrium favours the chloride-bound species and thus there remains a more aggregated state in solution even upon dilution. These results again suggest that the chloride-bound iron porphyrin species are most prone to aggregation.

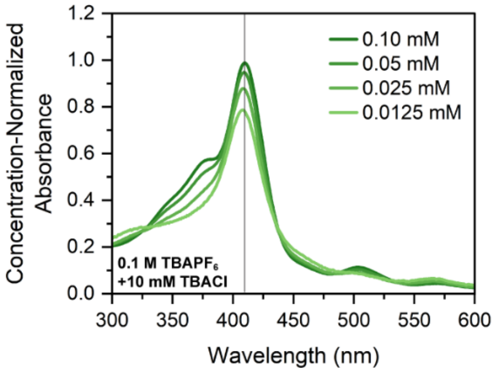

Figure S23. UV-Vis absorption spectra as a function of catalyst concentration under conditions of excess chloride at 0.10–0.0125 mM of **FeTriPP.** Conditions: indicated catalyst concentration, 0.1 M $TBAPF_6$ + 10 mM TBACl in DMF, 1 mm path length.

S12. Spectroelectrochemical Aggregation Studies

Spectroelectrochemical experiments were performed in an analogous manner to the previously presented concentration-normalized UV-Vis Q-Band aggregation studies, except here the reduced porphyrin species were investigated. The experimental setup consisted of a Cary 60 spectrophotometer fitted with a fiber optic coupler (Agilent Technologies, Inc.) and connected *via* fiber optic cables to a sample holder (OceanInsight) inside a nitrogen-filled glovebox, allowing for detection of the air-sensitive reduced porphyrin species. The electrochemical cell consisted of a 1 mm SEC-CT thin layer quartz glass cuvette, a platinum gauze flag working electrode (Bioanalytical Systems, Inc.), a platinum wire counter electrode (0.5 mm diameter), and a silver wire encased in a Vycor tip glass tube filled with 0.1 M $TBAPF_6$ electrolyte as the pseudo-reference electrode. Electrolysis was performed with a CHI650E potentiostat (CH Instruments, Inc.), where leads were connected to electrical feedthrough cables into the glovebox.

First, a concentrated solution of catalyst (1.0 mM) was prepared and an initial CV scan was taken (**Figure S24a,e**) to determine the applied potentials required to target each redox state of interest. Each potential was applied at a given catalyst concentration, where the applied potential was held with UV-Vis spectra being collected every 5 minutes for about 20-30 minutes until the spectra stabilized, indicating completion of the electrolysis. The catalyst solution was then diluted and a positive potential (~ +0.5 V) was applied to return to the formal Fe^{III} species before repeating the previous procedure at each catalyst concentration. We note that the concentration range tested in these spectroelectrochemical studies was limited due to scattering from the platinum gauze electrode resulting in a large baseline absorbance. For **FeTriPP** at the Fe(I) and Fe(II) redox states, (**Figure 24g,h**), the Q band spectra at 0.5 mM and 0.25 mM overlay, suggesting that aggregation is not significant below 0.5 mM. This is in contrast to the Fe(III) porphyrins, which show evidence of aggregation through Soret band shifts at much more dilute concentrations, suggesting less severe aggregation for reduced porphyrin species.

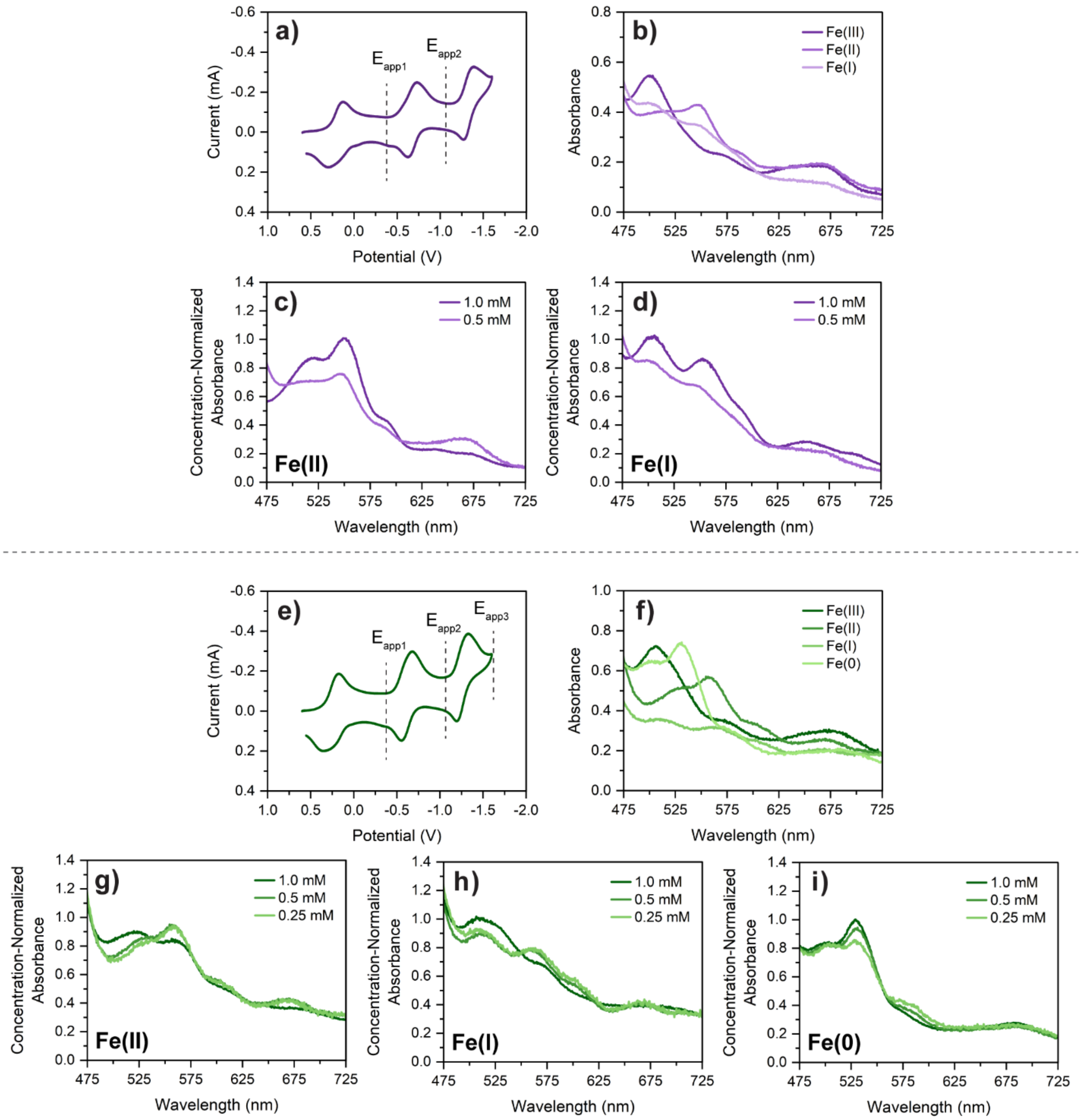

Figure S24. Spectroelectrochemical UV-Vis Q Band experiments. CVs of 1.0 mM (a) **FeDiPP** and (e) **FeTriPP**; E_{app1} = -0.45 V (~ -1.45 V vs. Fc/Fc^+), E_{app2} = -1.10 V (~ -2.10 V vs. Fc/Fc^+), E_{app3} = -1.6 V (~ -2.6 V vs. Fc/Fc^+). Representative stacked spectra of 0.5 mM (b) **FeDiPP** and (f) **FeTriPP** following electrolysis at each applied potential (E_{app}); species assigned to the formal Fe(II), Fe(I), and Fe(0) species for spectra at E_{app1}, E_{app2}, and E_{app3}, respectively. Concentration-normalized spectra following electrolysis at E_{app1} for (c) **FeDiPP** and (g) **FeTriPP**. Concentration-normalized spectra following electrolysis at E_{app2} for (d) **FeDiPP** and (h) **FeTriPP**. Concentration-normalized spectra following electrolysis at E_{app3} for (i) **FeTriPP**. Conditions: indicated catalyst concentration, 0.1 M $TBAPF_6$ in DMF, 1 mm path length.

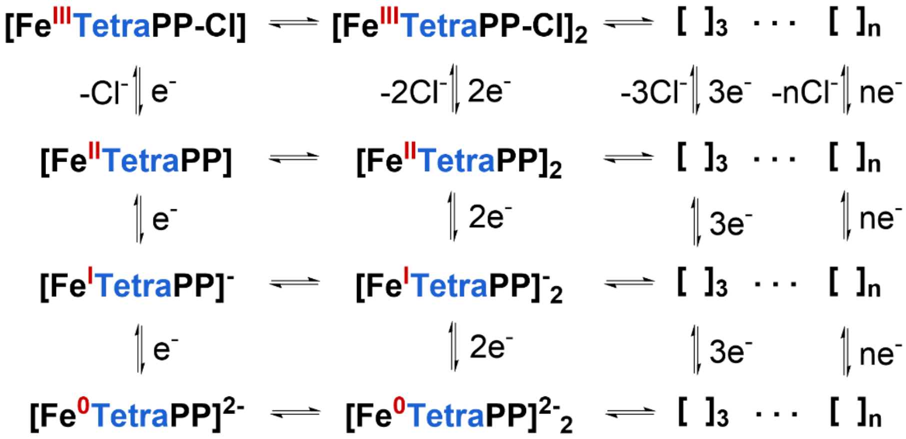

Figure S25. Schematic of proposed equilibria between monomeric, dimeric, and larger aggregates of iron porphyrins (horizontal equilibrium arrows) and electron transfers (vertical arrows) that highlights the solution speciation complexity. Additional speciation changes arising from axial ligand exchange or µ-oxo dimer are not depicted but likely also occur.

S13. Details of Computational Modeling of Porphyrin Aggregates

All calculations were performed using the Fritz Haber Institute -- ab initio materials simulations (FHI-aims) program.[14] They used the B86bPBE density functional,[15,16] the XDM dispersion correction,[17,18] the light basis setting, dense integration grids, and the atomic Zora scalar relativity correction.[5] The individual Fe complexes were assigned a high spin state with 5 unpaired electrons and their geometries fully optimized. We then constructed periodic chains of molecules, aligned in the c lattice direction and separated from neighbouring chains in the a,b directions by vacuum, with two molecules per unit cell and a fixed spin moment of 10. The a and b lattice vectors were kept fixed, while the c lattice vector and the atomic positions were allowed to optimize, using a 1x1x2 k-point mesh.

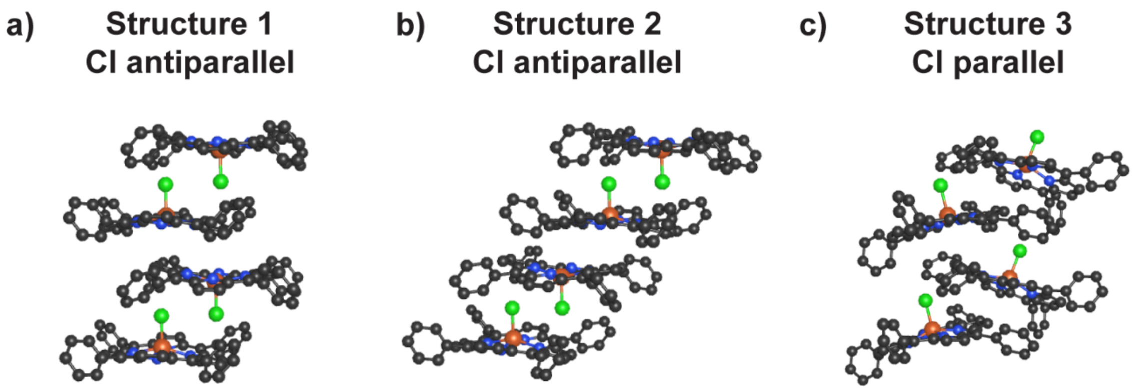

Figure S26. Iron(III) chloride porphyrin aggregate structures investigated, showing **FeTetraPP** as a representative case. Hydrogen atoms have been removed for clarity.

Table S3. Calculated aggregate binding energies and HOMO-LUMO energy gap shift for each different aggregate conformation and each catalyst structure.

	**Cl antiparallel**				**Cl parallel**	
	**Structure 1**		**Structure 2**		**Structure 3**	
Catalyst	Aggregate Binding Energy[a] (eV)	HOMO-LUMO Gap Shift (eV)	Aggregate Binding Energy[a] (eV)	HOMO-LUMO Gap Shift (eV)	Aggregate Binding Energy[a] (eV)	HOMO-LUMO Gap Shift (eV)
**FeDiPP**	-0.817	-0.040	-0.811	-0.038	-0.838	-0.090
**FeTriPP**	-0.936	-0.112	-0.935	-0.096	-1.035	-0.096
**FeTetraPP**	-1.088	-0.127	-1.071	-0.108	-1.099	-0.054

[a]Aggregate binding energy per molecule.

S14. Survey of Additional Metalloporphyrin Aggregation

S14.1 Survey of Substituted Iron Tetraphenylporphyrins

The UV-Vis Q band aggregation studies were repeated for some additional simple iron tetraphenylporphyin derivates. Due to reduced solubility of some of these complexes in DMF, the range of concentrations surveyed was adjusted as necessary. The free base porphyrin ligands were purchased from Frontier Scientific, and were metallated using the previously discussed procedure. The *para*-methoxy substituted iron tetraphenylporphyrin (FeT(*p*-OMe)PP) (**Figure S27a**) and the iron tetramesitylporphyrin (FeTMesP) (**Figure S27b**) both seem to show less severe spectral changes as a function of concentration, suggesting aggregation may be less severe for these porphyrins. However, the iron tetra(pentafluorophenyl)porphyrin ($FeF_{20}TPP$) (**Figure S27c**) shows similar concentration-dependent changes as the series of phenylporphyrins studied in this work. These results together suggest that catalyst structure can influence self-assembly behaviour, likely as a function of sterics on the *meso* positions of the porphyrin.

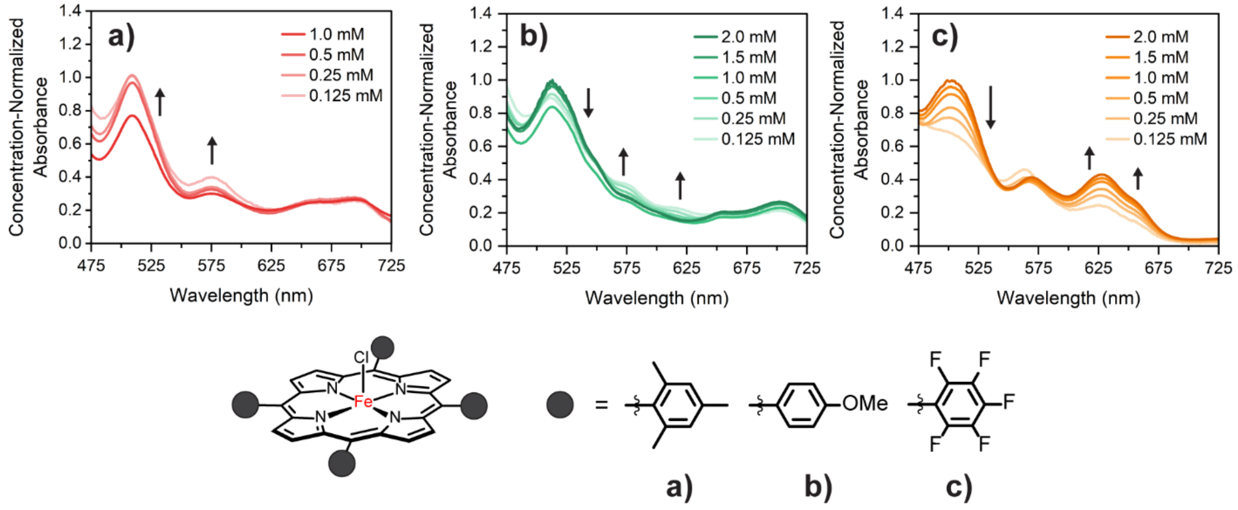

Figure S27. Concentration-dependent UV-Vis absorption spectra of substituted iron tetraphenylporphyrins (a) FeTMesP (b) FeT(*p*-OMe)PP (c) $FeF_{20}TPP$. Conditions: indicated concentration of catalyst, 0.1 M $TBAPF_6$ in DMF, 1 mm path length.

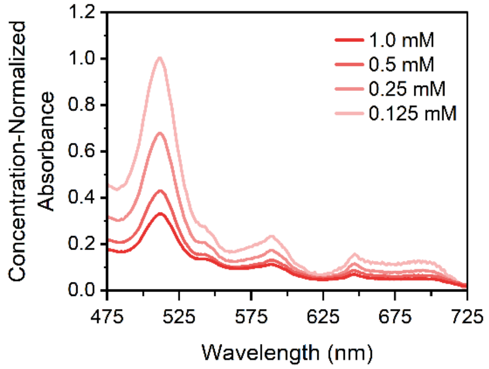

Figure S28. Concentration-dependent UV-Vis absorption spectra of FeTMesP in acetonitrile (MeCN) Conditions: indicated concentration of catalyst, 0.1 M $TBAPF_6$ in MeCN, 1 mm path length.

S14.2 Survey of Various Metallo-Tetraphenylporphyrins

The UV-Vis Q band aggregation studies were repeated for tetraphenylporphyrin complexes with various divalent metals. Due to reduced solubility of some of these complexes in DMF, the range of concentrations surveyed was adjusted as necessary. The free base tetraphenyl porphyrin ligand was purchased from Frontier Scientific, and was metalated using standard procedures. The cobalt, nickel, and copper tetraphenylporphyrins show evidence of aggregation with varying levels of severity, whereas the zinc tetraphenylporphyrin does not show any concentration-dependent spectral changes.

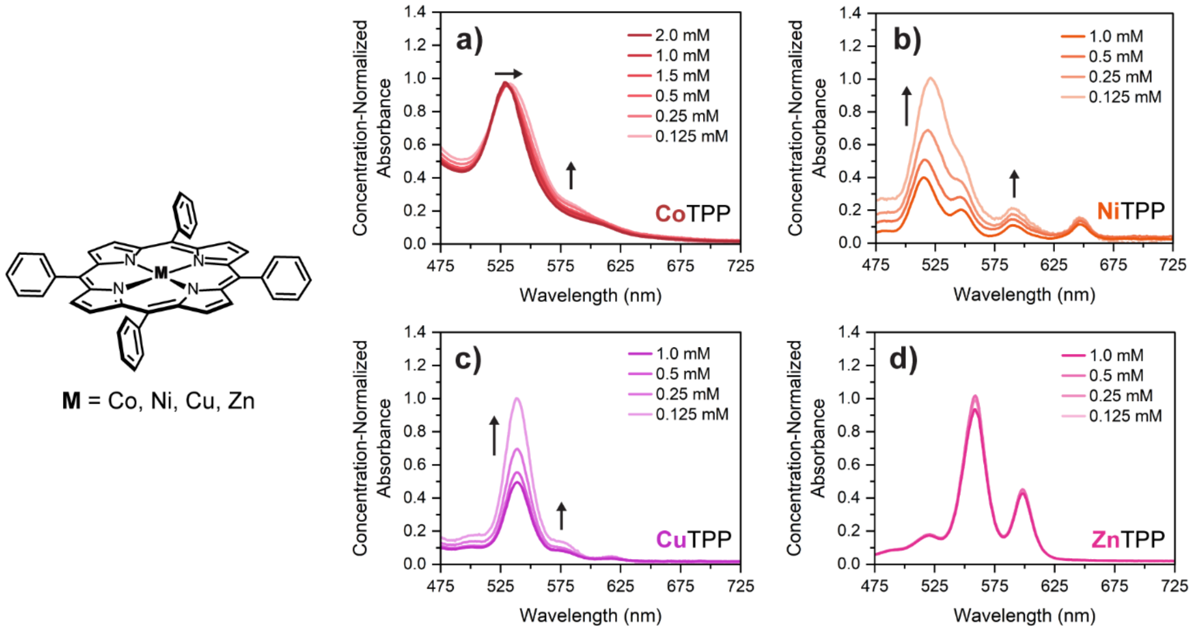

Figure S29. Concentration-dependent UV-Vis absorption spectra of various divalent metallo-tetraphenylporphyrins. (a) CoTPP (b) NiTPP (c) CuTPP (d) ZnTPP. Conditions: indicated concentration of catalyst, 0.1 M $TBAPF_6$ in DMF, 1 mm path length.

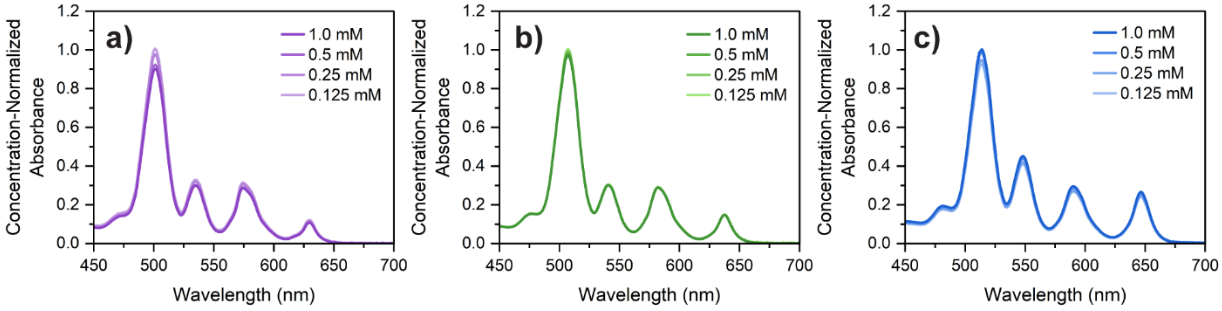

Figure S30. Concentration-dependent UV-Vis absorption spectra of free-base *meso*-phenyl porphyrin ligands. (a) DiPP (b) TriPP (c) TetraPP. Conditions: indicated concentration of porphyrin, 0.1 M $TBAPF_6$ in DMF, 1 mm path length.

S15. Spectra and Characterization

^{1}H NMR Spectra

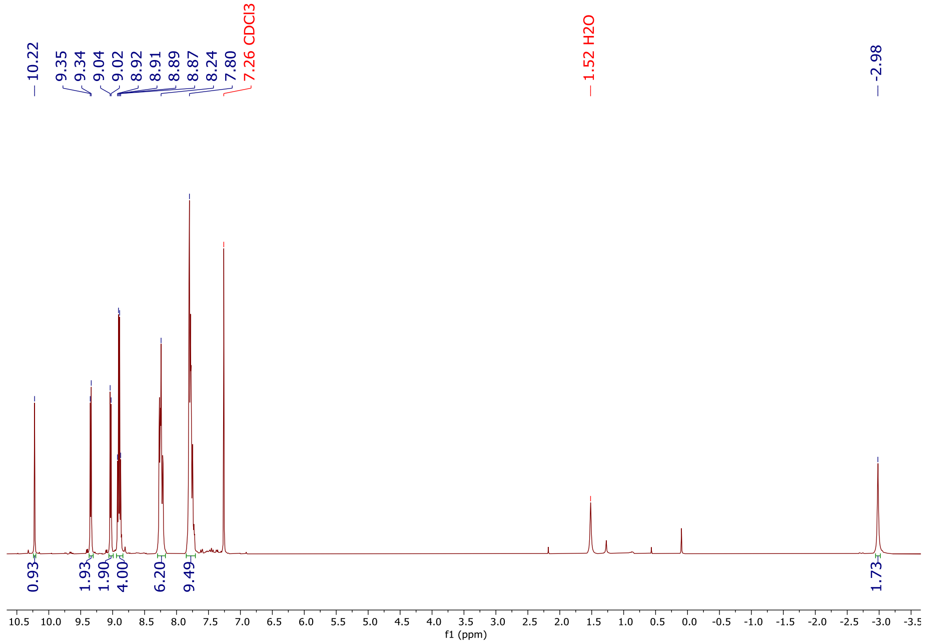

Figure S31. ^{1}H NMR (300 MHz) of TriPP in $CDCl_3$.

UV-Vis Spectra of Free-Base Ligands and Iron Complexes

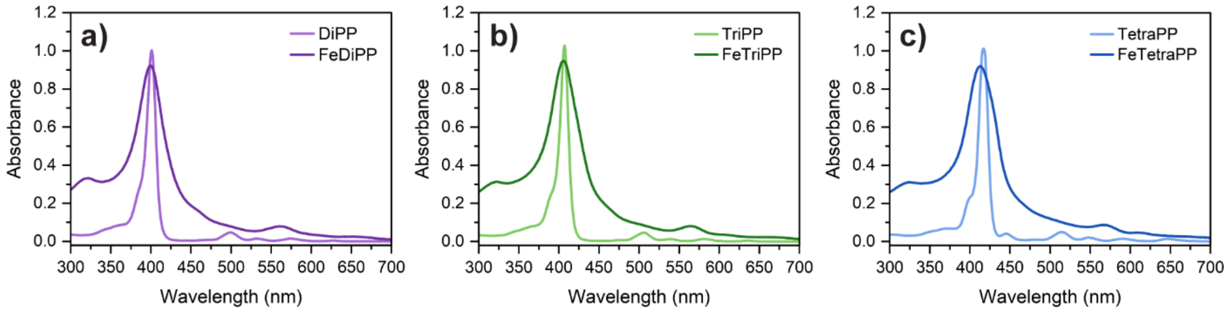

Figure S32. UV-Vis absorption spectra of free-base porphyrin ligand in MeOH (light traces) and metallated porphyrin complexes in DMF (dark traces); (a) **FeDiPP** (b) **FeTriPP** (c) **FeTetraPP**. Path length 1 cm.

ESI-MS of Final Complexes

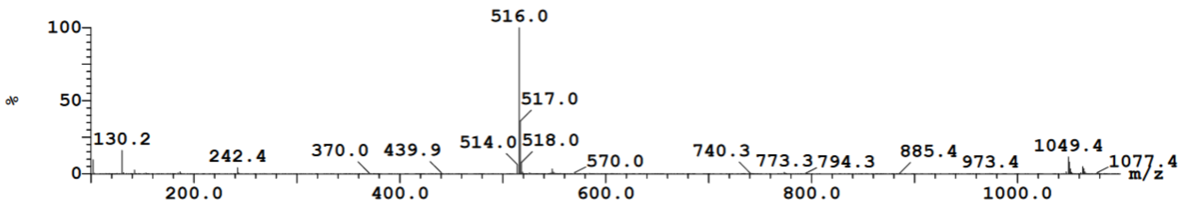

Figure S33. ESI-MS of *meso*-5,15-diphenylporphyrin iron(III) chloride (**FeDiPP**) in MeOH.

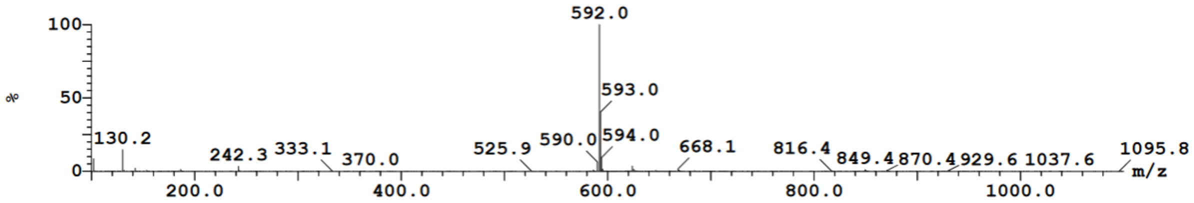

Figure S34. ESI-MS of *meso*-5,10,15-triphenylporphyrin iron(III) chloride (**FeTriPP**) in MeOH.

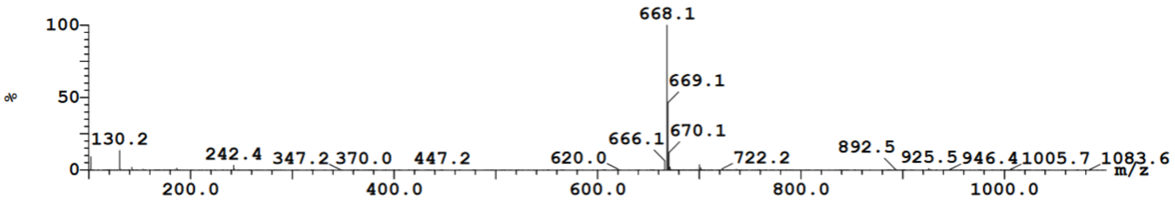

Figure S35. ESI-MS of *meso*-tetraphenylporphyrin iron(III) chloride (**FeTetraPP**) in MeOH.

Characterization of Surveyed Metalloporphyrins

FeT(*p*-Ome)PP. **MS (ESI)** observed (m/z): 788.5; calculated for $[M]^+$: 788.69. **UV-Vis** (DMF) λ_{max} (nm) 422, 579, 625.

FeTMesP. **MS (ESI)** observed (m/z): 836.6; calculated for $[M]^+$: 836.35. **UV-Vis** (DMF) λ_{max} (nm) 419, 510, 548, 575.

FeF$_{20}$TPP. **MS (ESI)** observed (m/z): 1128.6; calculated for $[M]^+$: 1127.98. **UV-Vis** (DMF) λ_{max} (nm) 420, 564.

CoTPP. **MS (ESI)** observed (m/z): 671.2; calculated for $[M]^+$: 671.16. **UV-Vis** (DMF) λ_{max} (nm) 414, 530.

NiTPP. **MS (ESI)** observed (m/z): 674.3; calculated for $[M]^+$: 671.43. **UV-Vis** (DMF) λ_{max} (nm) 415, 525, 590, 647.

CuTPP. **MS (ESI)** observed (m/z): 676.4; calculated for $[M]^+$: 675.16. **UV-Vis** (DMF) λ_{max} (nm) 414, 539.

ZnTPP. **MS (ESI)** observed (m/z): 677.3; calculated for $[M]^+$: 671.17. **UV-Vis** (DMF) λ_{max} (nm) 424, 519, 559, 598.

References

(1) Feng, X.; Senge, M. O. An Efficient Synthesis of Highly Functionalized Asymmetric Porphyrins with Organolithium Reagents. *J. Chem. Soc. Perkin 1* **2001**, No. 9, 1030–1038. https://doi.org/10.1039/b100012h.

(2) Nichols, E. M.; Derrick, J. S.; Nistanaki, S. K.; Smith, P. T.; Chang, C. J. Positional Effects of Second-Sphere Amide Pendants on Electrochemical CO_2 Reduction Catalyzed by Iron Porphyrins. *Chem. Sci.* **2018**, *9* (11), 2952–2960. https://doi.org/10.1039/c7sc04682k.

(3) Guo, K.; Li, X.; Lei, H.; Zhang, W.; Cao, R. Unexpected Effect of Intramolecular Phenolic Group on Electrocatalytic CO_2 Reduction. *ChemCatChem* **2020**, *12* (6), 1591–1595. https://doi.org/10.1002/cctc.201902034.

(4) Wasylenko, D. J.; Rodríguez, C.; Pegis, M. L.; Mayer, J. M. Direct Comparison of Electrochemical and Spectrochemical Kinetics for Catalytic Oxygen Reduction. *J. Am. Chem. Soc.* **2014**, *136* (36), 12544–12547. https://doi.org/10.1021/ja505667t.

(5) Passard, G.; Dogutan, D. K.; Qiu, M.; Costentin, C.; Nocera, D. G. Oxygen Reduction Reaction Promoted by Manganese Porphyrins. *ACS Catal.* **2018**, *8* (9), 8671–8679. https://doi.org/10.1021/acscatal.8b01944.

(6) Derrick, J. S.; Loipersberger, M.; Nistanaki, S. K.; Rothweiler, A. V.; Head-Gordon, M.; Nichols, E. M.; Chang, C. J. Templating Bicarbonate in the Second Coordination Sphere Enhances Electrochemical CO_2 Reduction Catalyzed by Iron Porphyrins. *J. Am. Chem. Soc.* **2022**, *144* (26), 11656–11663. https://doi.org/10.1021/jacs.2c02972.

(7) Teindl, K.; Patrick, B. O.; Nichols, E. M. Linear Free Energy Relationships and Transition State Analysis of CO_2 Reduction Catalysts Bearing Second Coordination Spheres with Tunable Acidity. *J. Am. Chem. Soc.* **2023**, *145* (31), 17176–17186. https://doi.org/10.1021/jacs.3c03919.

(8) Costentin, C.; Drouet, S.; Robert, M.; Savéant, J. M. Turnover Numbers, Turnover Frequencies, and Overpotential in Molecular Catalysis of Electrochemical Reactions. Cyclic Voltammetry and Preparative-Scale Electrolysis. *J. Am. Chem. Soc.* **2012**, *134* (27), 11235–11242. https://doi.org/10.1021/ja303560c.

(9) Lee, K. J.; Elgrishi, N.; Kandemir, B.; Dempsey, J. L. Electrochemical and Spectroscopic Methods for Evaluating Molecular Electrocatalysts. *Nat. Rev. Chem.* **2017**, *1* (0039). https://doi.org/10.1038/s41570-017-0039.

(10) Pegis, M. L.; McKeown, B. A.; Kumar, N.; Lang, K.; Wasylenko, D. J.; Zhang, X. P.; Raugei, S.; Mayer, J. M. Homogenous Electrocatalytic Oxygen Reduction Rates Correlate with Reaction Overpotential in Acidic Organic Solutions. *ACS Cent. Sci.* **2016**, *2* (11), 850–856. https://doi.org/10.1021/acscentsci.6b00261.

(11) Lexa, D.; Rentien, P.; Savéant, J. M.; Xu, F. Methods for Investigating the Mechanistic and Kinetic Role of Ligand Exchange Reactions in Coordination Electrochemistry. Cyclic Voltammetry of Chloroiron(III)Tetraphenylporphyrin in Dimethylformamide. *J. Electroanal. Chem.* **1985**, *191* (2), 253–279. https://doi.org/10.1016/S0022-0728(85)80021-8.

(12) Leaist, D. G. The Effects of Aggregation, Counterion Binding, and Added NaCl on Diffusion of Aqueous Methylene Blue. *Can. J. Chem.* **1988**, *66* (9), 2452–2457. https://doi.org/10.1139/v88-386.

(13) Branson, H. Diffusion as a Function of Aggregation in Colloidal Media. *Bull. Math. Biophys.* **1942**, No. 4, 1–6. https://doi.org/10.1007/BF02477349.

(14) Blum, V.; Gehrke, R.; Hanke, F.; Havu, P.; Havu, V.; Ren, X.; Reuter, K.; Scheffler, M. Ab Initio Molecular Simulations with Numeric Atom-Centered Orbitals. *Comput. Phys. Commun.* **2009**, *180* (11), 2175–2196. https://doi.org/10.1016/j.cpc.2009.06.022.

(15) Becke, A. D. On the Large-Gradient Behavior of the Density Functional Exchange Energy. *J. Chem. Phys.* **1986**, *85* (12), 7184–7187. https://doi.org/10.1063/1.451353.

(16) Perdew, J. P.; Burke, K.; Ernzerhof, M. Generalized Gradient Approximation Made Simple. *Phys. Rev. Lett.* **1996**, *77* (18), 3865–3868. https://doi.org/10.1103/PhysRevLett.77.3865.

(17) Price, A. J. A.; Otero-de-la-Roza, A.; Johnson, E. R. XDM-Corrected Hybrid DFT with Numerical Atomic Orbitals Predicts Molecular Crystal Lattice Energies with Unprecedented Accuracy. *Chem. Sci.* **2022**, *14* (5), 1252–1262. https://doi.org/10.1039/d2sc05997e.

(18) Johnson, E. R. The Exchange-Hole Dipole Moment Dispersion Model. In *Non-Covalent Interactions in Quantum Chemistry and Physics: Theory and Applications*; Otero-de-la-Roza, A., DiLabio, G. A., Eds.; Elsevier, 2017; pp 169–194. https://doi.org/10.1016/B978-0-12-809835-6.00006-2.
